# Four IgG Antibodies and Protein G Are Shapeshifters

**DOI:** 10.3390/ijms27177662

**Published:** 2026-08-26

**Authors:** Michael O. Glocker, Manuela Ruß, Cornelia Koy, Michael Kreutzer, Fiona T. I. Melder, Yelena Diebler, Harald Illges, Kwabena F. M. Opuni

**Affiliations:** 1 Proteome Center Rostock, Medical Faculty and Natural Science Faculty, University of Rostock, Schillingallee 69, 18057 Rostock, Germany; 2 Department of Applied Natural Sciences, Immunology and Cell Biology, Institute of Functional Gene Analytics, University of Applied Sciences Bonn-Rhein-Sieg, von-Liebig-Str. 20, 53359 Rheinbach, Germany; harald.illges@h-brs.de; 3 Department of Pharmaceutical Chemistry, School of Pharmacy, College of Health Science, University of Ghana, Legon P.O. Box LG43, Ghana; kfopuni@ug.edu.gh; 4 Food and Drugs Authority Ghana, Cantonments, Accra P.O. Box CT2783, Ghana

**Keywords:** opsonophagocytosis, IgG, protein G, nanoESI-MS, ion mobility mass spectrometry, collision cross section, biocomputational analysis, molecular modeling, I-TASSER, Pepfold4, AlphaFold3

## Abstract

Studying protein structure dynamics is key to understanding protein function modulation. Alternative protein conformations are well discriminated from each other by nanoESI mass spectrometry and ion mobility measurements. Experimentally determined collisional cross-sections were compared to calculated collisional cross-sections of fifteen peptides, single-domain proteins, and protein complexes. The multi-domain proteins investigated here, four immunoglobulin G (IgG) antibodies and protein G, are present as compacted/folded “native” conformations in neutral buffered solutions, and they are identified by molecular ions with narrow charge-state distributions, relatively few charges, and small collisional cross-sections. Simultaneously present extended/folded but nevertheless “native” conformations produced additional ions with higher charge states, different charge-state distributions, and larger collisional cross-sections. Computed collisional cross-sections from compacted “o-shape” and extended “l-shape” protein G three-dimensional (3D) structures match experimental data, indicating equilibrium, and suggest a dynamic “o2l” flip process. Likewise, “m-shape” (compacted) and “Y-shape” (extended) IgGs are regarded as two supposedly reversibly adopted antibody conformations which may interchange by an “m2Y” flip. Adopting an m-shape would prevent an antibody-based initiation of humoral and cellular immune system responses, such as opsonophagocytosis, prior to antigen contact, which stands in line with the rearrangement hypothesis.

## 1. Introduction

Opsonophagocytosis is a fundamental mechanism through which pathogens are removed upon antibody binding, e.g., by neutrophils and macrophages. The still-unsolved question of how opsonophagocytosis is regulated at the molecular level has divided the scientific community [1]. The associative hypothesis proposes accumulation of crystallizable fragment (Fc) regions by multiple antibodies binding on a pathogen’s surface as a precondition to increase the avidity of Fc receptor interactions [2], thereby triggering cellular signal-transduction cascades. By contrast, the conformational rearrangement hypothesis in response to antigen binding allows a direct increase in affinity between an antibody’s Fc region and the Fc receptor [3], placing antibody conformational changes at the focus of downstream cellular signaling events.

Protein conformations collectively describe differing higher-order protein structures without changes in covalent atom bonds [4]. Alterations in protein conformation include subtle changes, such as locally restrained bond-angle movements of a few atom bonds, e.g., affecting amino acid side chain interactions at the catalytic center of an enzyme [5]. And on a more global scale, conformational changes encompass domain–domain re-orientation, which might alter macromolecular interactions by opening up otherwise-hidden binding surfaces, as is the case with reversible actin–myosin binding in muscle fibers [6]. Hence, while defined structures are assumed by proteins to ascertain distinct functions, protein structure dynamics is a prerequisite for protein function modulation [7].

Methods which provide information on protein structure dynamics often focus on specific reporter units, e.g., chromophores, which are either chemically added [8,9] or intrinsically present [10] and for which their property changes are monitored while the investigated protein responds to a changing (micro)environment, e.g., by changing its structure. The in-silico approach for obtaining molecular dynamics information has become quite powerful and, in principle, provides holistic molecular structure information [11]. Its limitations are (i) the necessity to translate assumed experimental conditions as best as possible into computer models and (ii) the as-yet very short accessible dynamics time scales [12,13].

“Native mass spectrometry” has shown that electrospray ionization (ESI)-generated narrow charge-state distributions with relatively few charges are typically obtained from protein solutions in which proteins adopt compact conformations [14,15,16]. The generally accepted correlation between charge-state distribution and compactness of protein conformation has been established already in the early days of protein ESI mass spectrometry [17,18]. Ion mobility mass spectrometry, a rather novel addition to the toolbox of methods which are capable of observing molecular-structure-associated features, including large-scale dynamic protein structure changes [19,20], allows for an independent dataset of characteristic protein structure features to be collected. Measuring drift times of (multiply protonated) molecular ions in the gas phase has become useful for estimating sizes of small molecules, as well as of large bio-macromolecules and protein complexes [21,22,23]. And collisional cross-sections (CCSs) determined from the drift times proved highly reproducible and fairly precise [24]. For example, the gas-phase CCS values for the monoclonal antibody benralizumab were found to stand in good agreement with those from measurements of the NIST monoclonal antibody [25,26]. Moreover, gas-phase CCSs are considered valuable measures of molecular dimensions of in-solution protein structures. Intriguingly, computed CCSs from 3D structures of proteins and protein complexes [27,28,29] have been found to match well [30] within acceptable tolerances [31].

With the aim of better understanding activation/deactivation of molecular function by protein structure alteration, we studied experimental and computational CCSs from two distinct multi-domain proteins: protein G’e (an N-terminally extended protein G version) and IgG. Both protein types exhibited two or more charge-state distributions in their ESI mass spectra. Charge-state envelopes with lower charges followed a linear CCS-to-molecular mass correlation observed for other compactly folded single-domain proteins. By contrast, the higher charge-state envelopes of protein G’e, as well as those of all studied IgGs, deviated significantly from this correlation. Because of the fact that these larger CCSs from the extended molecular structures differed from those of the denatured proteins, they must still be considered to belong to “native” protein conformations, indicating the shapeshifting behavior of protein G’e and IgGs at neutral pH. Three-dimensional molecular structures or structural models with either compact or extended protein conformations were used to compute smaller and larger CCSs for protein G’e and IgGs, respectively. The well-matching experimental and computational CCSs lend evidence to the assumption that the two distinguishable “native” conformations of either protein G’e or IgGs can be simultaneously present in neutral pH solutions, forming in-solution equilibria of interchanging conformations and thereby enabling modulation of protein functions.

## 2. Results

### 2.1. Peptides, Single-Domain Proteins, and Complexes

Collisional cross-sections (CCSs) of five peptides, seven single-domain proteins, and three protein complexes (for amino acid sequences see Table S1) were calculated using 3D structures, i.e., atom coordinate files which were downloaded from the Research Collaboratory for Structural Bioinformatics (RSCB) protein data bank. In cases where there were no solved 3D structure coordinates available, suitable 3D structures were in-silico modeled using online modeling software tools, such as PepFold4 or I-TASSER (see Table S2). For determining computed CCSs, the 3D structure files were submitted to the Collidoscope software, and charge states were selected to match the most abundant charge states recorded in the mass spectra of the respective peptides, single-domain proteins, and complexes (Figures S1–S15). The computed CCSs spanned a range from 259 Å^2^ for doubly protonated bradykinin to 8430 Å^2^ for alcohol dehydrogenase with 25 attached protons (Table S2).

In addition to measuring the *m*/*z* values of all peptides, proteins, and complexes by nanoESI mass spectrometry under neutral pH conditions (“native” mass spectrometry), the ion mobility drift times of the most abundant ion signals were recorded in duplicate (Figures S1–S15, Tables S3 and S4). These drift times were used to determine experimental CCSs (Table S4), which ranged from 331 ± 0 Å^2^ for doubly protonated bradykinin to 7577 ± 26 Å^2^ for alcohol dehydrogenase with 25 added protons.

**Figure 1:**
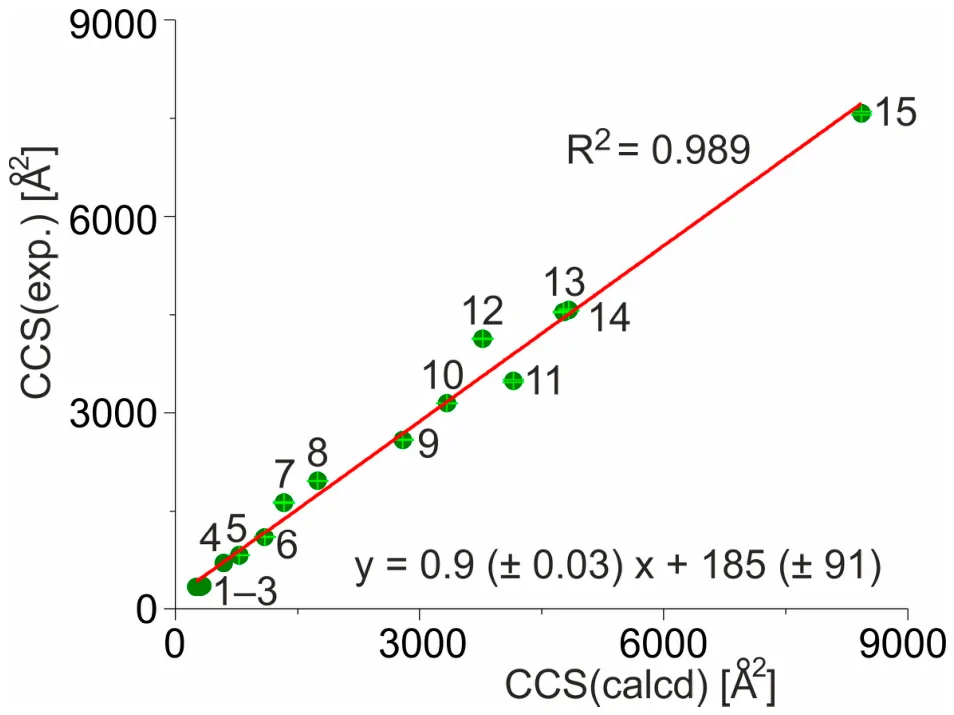
Experimental CCS values over computed CCS values of peptides, single-domain proteins, and complexes. 1: Angiotensin II. 2: Bradykinin. 3: Angiotensin I. 4: Mellitin. 5: Insulin. 6: Ubiquitin. 7: Cytochrome C. 8: Holomyoglobin. 9: Pepsin. 10: Ovalbumin. 11: IgG-Fc. 12: Streptavidin. 13: Hemoglobin. 14: Serum albumin. 15: Alcohol dehydrogenase. For individual charge states and CCS values, see Table S5.

The remarkable correlation between computed and measured CCS values is readily observed when plotting experimental CCSs (average values) over computed CCSs (Figure 1, Table S5), which leads to a linear regression line with a slope of 0.9 (±0.03) and a y-axis intercept of 185 (±91). This good correlation between computed and experimental CCS values holds as long as 3D structures or structure models represent compactly folded molecules and/or complexes which contain compactly folded protein units and as long as experimental determinations of CCS values are performed under “native” mass spectrometry conditions. Note that this is also true for pepsin, for which “native” mass spectrometry conditions are assumed at around pH 2.

With the confirmation that ion mobility mass spectrometry enables experimental determination of CCS values in the gas phase which stand in excellent agreement with those computed from 3D structures of compactly folded proteins in condensed phase, we investigated whether or not experimentally determined CCSs provided accurate information for multi-domain proteins, particularly protein G’e and IgGs, which may adopt more than one conformation in buffered solutions at neutral pH.

### 2.2. Protein G’e

**Figure 2:**
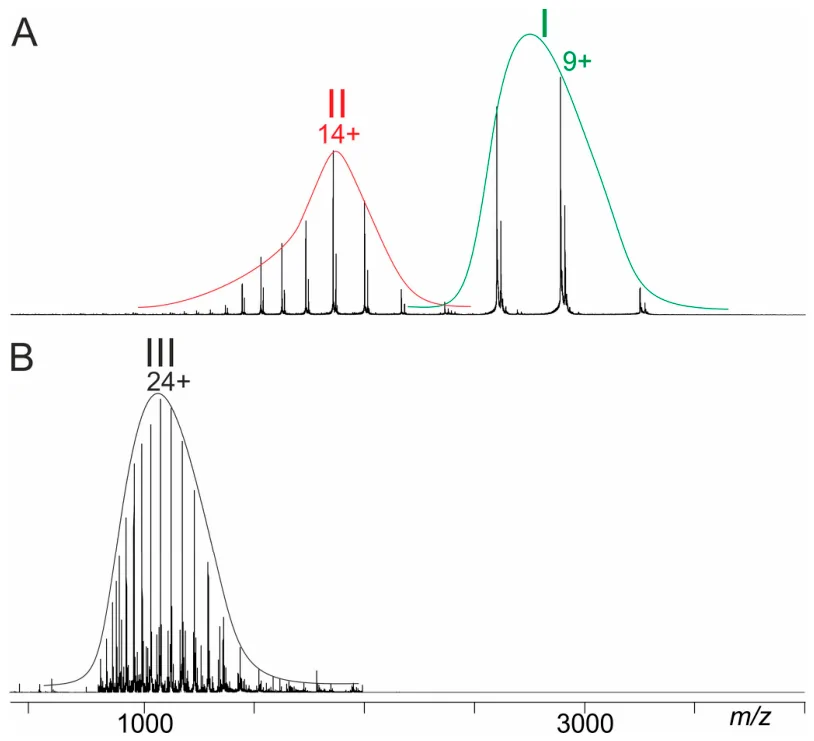
NanoESI mass spectra of Protein G’e (A1) dissolved in neutral and acidic solutions. (**A**): 200 mM ammonium acetate, pH 7. (**B**): 10% acetic acid/methanol (9:1, *v*/*v*), pH 2. Charge states of selected multiply protonated ion signals are given. Distinguishable charge-state distributions are enveloped and labeled with Roman numerals. Green: Compacted native conformation (I). Red: Extended native conformation (II). Black: Denatured conformation (III).

First, we analyzed mass spectra of protein G’e (A1; for amino acid sequences, see Table S6), for which we had previously established the following: (i) This protein consists of three identical domains which are held together by two linker sequences, and (ii) at neutral pH, it adopts two clearly distinguishable in-solution conformations. These two readily distinguishable in-solution conformations instantly reveal themselves via “native” mass spectrometry because, in the ESI mass spectrum, there are two separate charge-state distributions displayed, with one centering on the 9+ ion signal and the other on the 14+ ion signal (Figure 2). Of note, these two distinct charge-state distributions are noticeably different from a third charge-state distribution, with the most abundant ion signal being the 24-fold protonated ion, which is recorded when protein G’e is dissolved in denaturing solvents at pH 2. In addition to determining the protein G’e molecular mass from the recorded ion signals (Table S7), ion mobility measurements were performed, and these afforded CCSs of each of the ions (Figure S16). As expected, the experimentally determined CCSs of the multiply protonated ions increased with charge states, where the 9+ ion signal shows a CCS value of 2260 ± 2 Å^2^ as opposed to the CCS value of 3403 ± 0 Å^2^ for the 14+ ion signal. Assuming that the 9+ ion signal represents compacted/folded protein G’e, whereas the 14+ ion signal represents an extended/folded protein G’e conformer, has become the general consensus. It should be noted that the CCS values for denatured protein G’e ion signals, e.g., the 24+ ion signal at 5742 ± 0 Å^2^, are much larger than those of the other two conformations (Table 1 and Tables S8 and S9).

Since there are two different 3D structure models available for protein G’e, one with a compacted/folded conformation (I-TASSER model) and one with an extended/folded conformation (AlphaFold3 model), CCS values for either of them had been computed (Table 1 and Table S10). As with single-domain proteins, we find a remarkable correlation between computed CCS values from the modeled protein G’e 3D structures and the mass spectrometry experimentally determined CCSs of either of the two major protein G’e conformations which were simultaneously recorded under “native” mass spectrometry conditions.

### 2.3. IgG Immunoglobulins

**Figure 3:**
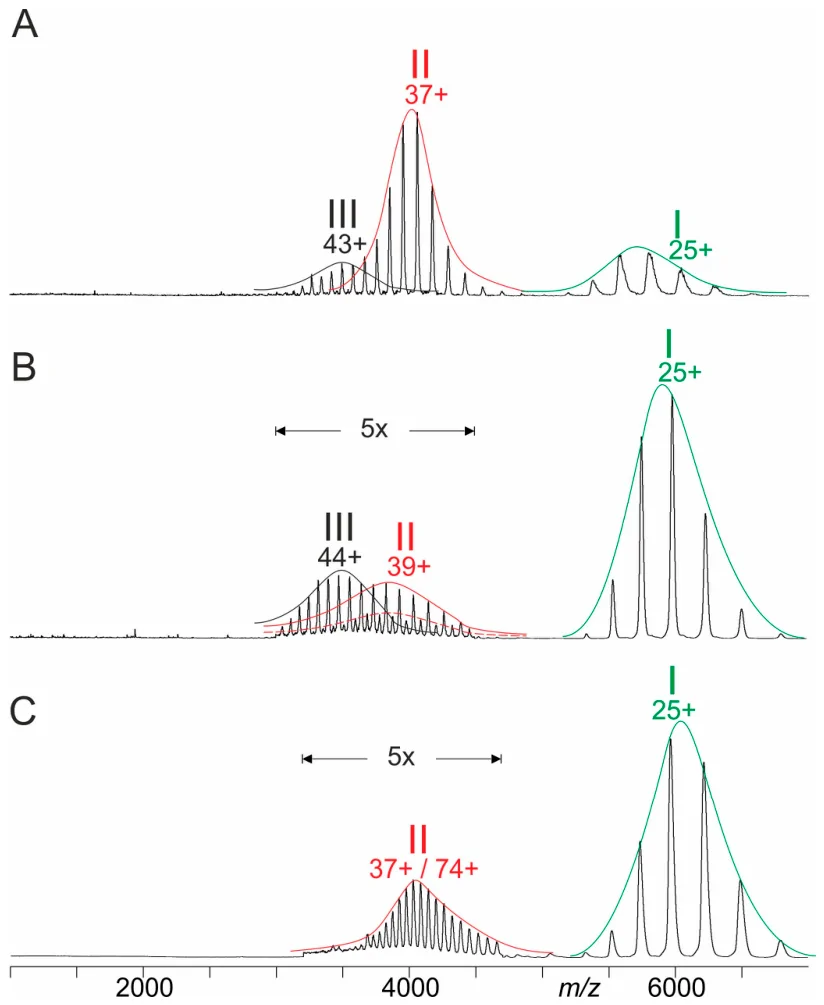
NanoESI mass spectra of antibodies dissolved in neutral solutions. (**A**): HAM1101 (A2α). (**B**): αMSP1_19_ (A2β). (**C**): αHis-tag (A2γ) antibody. Solvent: 200 mM ammonium acetate, pH 7. Charge states of selected multiply protonated ion signals are given. Distinguishable charge-state distributions are enveloped and labeled with Roman numerals. Green: Compacted native conformation (I). Red: Extended native conformation (II). Black: Denatured conformation (III).

Having substantiated that ion mobility mass spectrometry provides insights into distinguishable conformations of the multi-domain protein G’e, we focused on studies of IgG immunoglobulins. We investigated four different IgG immunoglobulins, HAM1101 (A2α), αMSP1_19_ (A2β), αHis-tag (A2γ), and Rituximab (A2δ), one after the other using “native” mass spectrometry conditions, and all four antibodies provided very similar results. IgG immunoglobulins are multi-domain proteins which, when recorded under “native” conditions, show well-known characteristic ion signals in ESI mass spectra with rather narrow charge-sstate distributions, centering on the 25-fold protonated quasi-molecular ion as the most intense ion signal of this ion series (Figure 3 and Figure S17–S20). Analyses of ion mobility ESI mass spectra of IgG enabled simultaneous determination of molecular masses (Table S7) and drift times (Tables S11–S13). For all recorded ions, the respective CCSs were calculated (Table 2).

In addition to the commonly observed ion charge-state distribution, we observed additional ion series, in most cases with lower abundances, with the most intense ion signals at approximately 37+ or 38+ charges under “native” mass spectrometry conditions. In some cases, the presence of non-covalent IgG dimers with approximately 74+ charges was recorded. On top of this, a third charge-state distribution series with the most intense ion at 44+ charges or higher was sometimes observed for monomeric IgG (Figure 3). This latter high-charge-state ion distribution pattern was reminiscent of the ion charge structures which were obtained when IgGs were investigated by ESI mass spectrometry under denaturing conditions at pH 2 (Figures S21 and S22).

Of note, the experimentally determined CCSs of the 25+ IgG ion signals in all four investigated IgG representatives resulted in CCS values of about 7500 Å^2^, which is much lower than the computed CCS value of 9948 Å^2^ (Table 2 and Table S10) obtained from the IgG X-ray structure (1IGY.pdb file deposited in the RSCB protein data bank). It should be noted that the IgG X-ray structure represents an extended/folded conformation of the multi-domain IgG immunoglobulin. Interestingly, the CCS value of around 10,000 Å^2^, which is experimentally determined for the 37+ or 38+ ion signals by ion mobility mass spectrometry under “native” ESI mass spectrometry conditions, fits well to the computed CCS value of 9948 Å^2^ using the X-ray-analysis-derived 3D structure. Based on all assembled experience, the 37+ or 38+ ion signals of the four investigated antibodies are tentatively assigned to represent extended/folded IgG conformations, known as the familiar “Y-shaped” IgG structures. Likewise, the CCS value of the non-covalent IgG dimer ions with 74+ charges was determined to be 20,177 ± 71 Å^2^, which fits a dimer that consists of two extended/folded (Y-shaped) IgGs. Consequently, we deduced that the 25+ ion signals represent compacted/folded IgGs with structures that differ from those obtained using X-ray crystallography data. In fact, from an “m-shaped” IgG model structure, one computes a collisional cross-section of 7291 Å^2^ (Table 2), which stands in excellent agreement with the nanoESI mass-spectrometry-measured CCS values of the 25+ ion signals of compacted/folded IgGs.

### 2.4. Correlation Between Protein Conformation and Molecular Mass

The striking linear dependency between the CCSs of 3D structures/structure models and those experimentally determined via ion mobility mass spectrometry for compactly folded “native” proteins—i.e., after recording multiply protonated molecular ions under “native” mass spectrometry conditions—led to the assumption that it might be possible to also observe a linear dependency between experimentally determined CCSs and the molecular masses of proteins/complexes. And indeed, plotting the experimentally determined CCSs obtained by ion mobility mass spectrometry against molecular mass showed a rather good linear dependency relationship, with only a marginal kink at about 20 kDa, which is likely the result of two independent calibrations (Figure S23).

**Figure 4:**
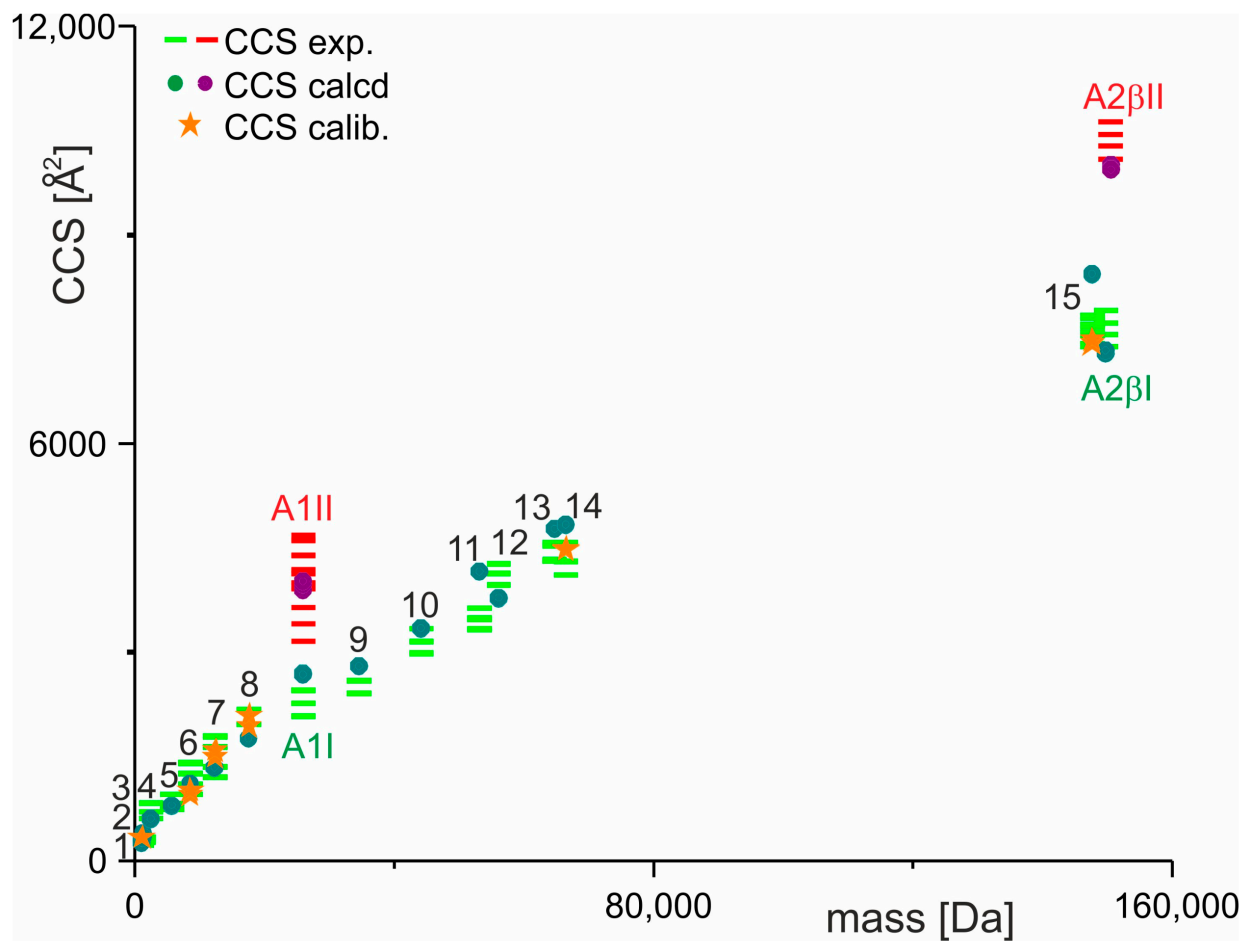
Collisional cross-section with respect to molecular mass of peptides, proteins, and complexes. 1: Angiotensin II. 2: Bradykinin. 3: Angiotensin I. 4: Mellitin. 5: Insulin. 6: Ubiquitin. 7: Cytochrome C. 8: Myoglobin. 9: Pepsin. 10: Ovalbumin. 11: IgGFc. 12: Streptavidin. 13: Hemoglobin. 14: Serum albumin. 15: Alcohol dehydrogenase. A1: Protein G’e. A2β: αMSP1_19_. Horizontal lines (green or red) show experimental values for different charge states. Filled circles (dark green or dark red) represent computed values. Orange asterisks indicate values used for calibration. Green: Compacted native conformations (I). Red: Extended native conformations (II). For numerical entries, see Tables S2, S4 and S8–S10.

Interestingly, the CCSs of the compacted/folded “native” conformations of the two shapeshifting multi-domain proteins, protein G’e (“o-shaped” according to our interpretation; A1I) and all investigated IgGs (“m-shaped” according to our interpretation; A2xI), fit well within the noted linear dependency between CCS and molecular mass (Figure 4). Moreover, this linear dependency is also true for CCS values computed from 3D structures and for the CCS values which were reported in the literature. By contrast, the CCSs of the extended/folded conformations of the two shapeshifting multi-domain protein types—protein G’e (“l-shaped” according to our interpretation; A1II) and IgGs (“Y-shaped” according to our interpretation; A2xII), which both must be considered “native” as well—substantially deviated from the observed linear dependencies.

## 3. Discussion

**Figure 5:**
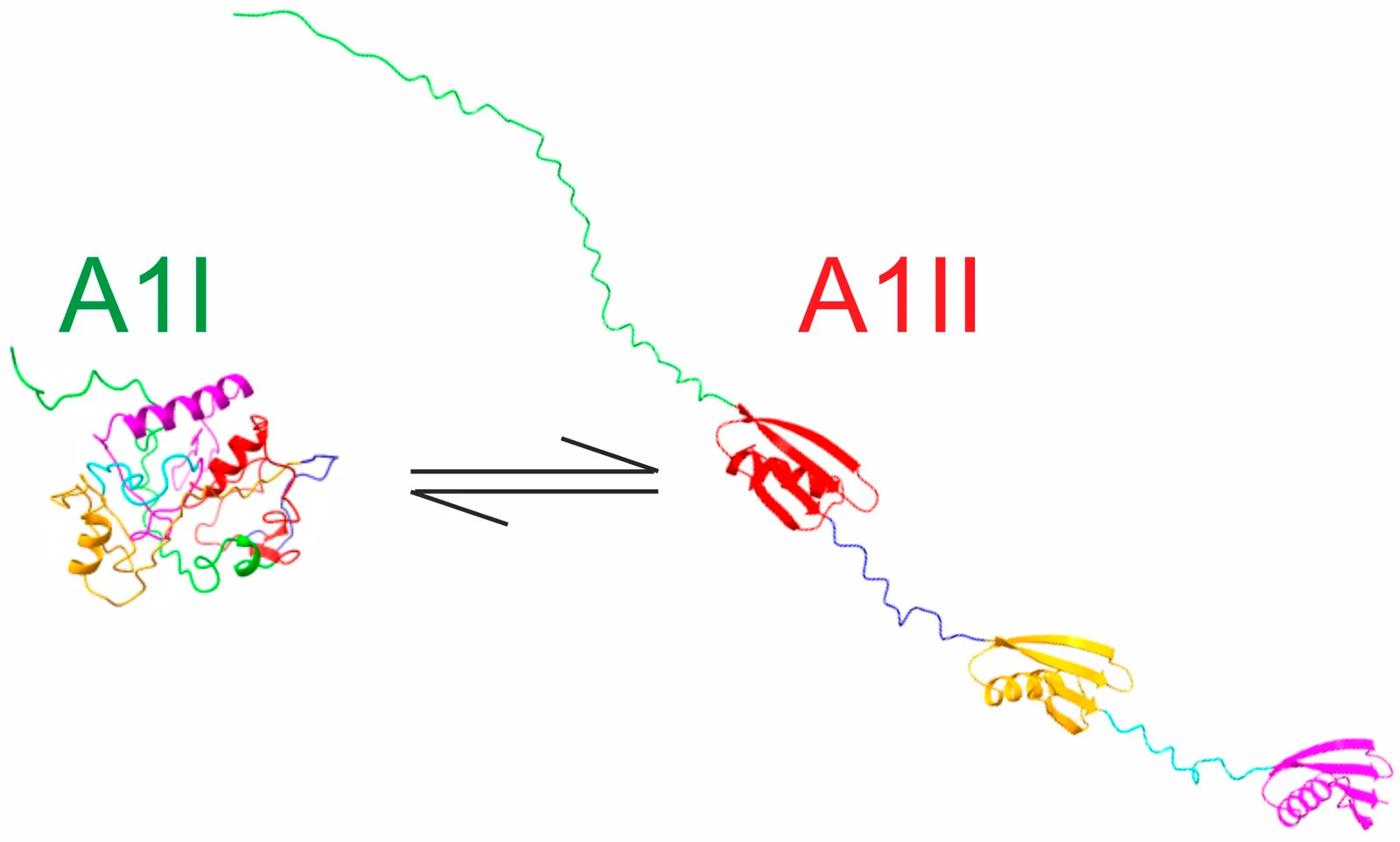
Equilibrium of protein G’e conformers. Left: “o-shaped” compacted conformation (A1I; I-TASSER structure model). Right: “l-shaped” extended conformation (A1II; AlphaFold3 structure model).

Because of the simultaneous presence of two differing charge-state distributions for protein G’e upon electrospraying from a neutral pH solution, we postulate that the underlying molecules adapted two “native” conformations and are present in solution in equilibrium (Figure 5).

Under neutral pH conditions, protein G binds IgG. Protein G’s IgG binding capability is lost at acidic pH but can be restored by re-adjusting pH to neutral, which is consistent with pH-induced reversible conformational changes of differing protein G structures.

**Figure 6:**
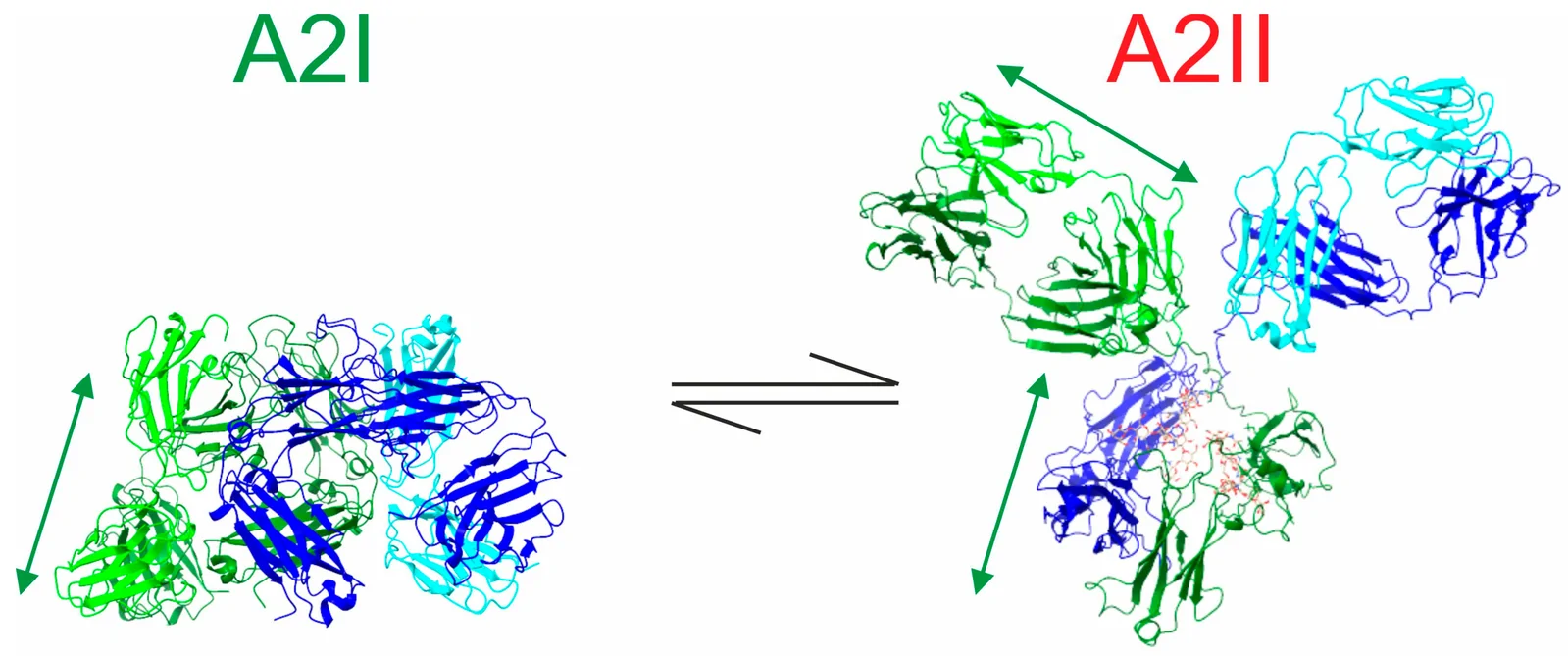
Equilibrium of IgG immunoglobulin conformations. Left: “m-shaped” compacted conformation (A2xI; Hojrup model). Right: “Y-shaped” extended conformation (A2xII; 1IGY.pdb). The green double-headed arrows are of equal lengths and are shown for size estimation of antigen binding fragment (Fab) and Fc domains, respectively. Heavy chains: dark blue or dark green. Light chains: light blue or light green.

Likewise, based on our results, we state that “native” nanoESI mass spectrometry is capable of simultaneously collecting charge-state distributions from distinct “native” IgG conformations. Again, we postulate that “m-shaped” and “Y-shaped” conformers form in-solution equilibria (Figure 6). Consequently, we postulate that the “compact” IgG conformations of all four investigated antibodies mirror “native m-shaped” IgG in-solution conformations.

The widespread conception that “Y-shaped” antibody conformations are (solely) present in the condensed phase is deeply ingrained in textbooks. But there are merely a handful of full-length IgG X-ray structures available in the Protein Data Bank. Most often mentioned are the IgG X-ray structures 1IGT, 1IGY, 1HZH, and 5DK3 [32]. Two structures, 1IGT (IgG2) and 1IGY (IgG1), which are depicted in most textbooks, were published by the same group [33,34]. From the others, 5DK3 (IgG4) was engineered to carry modified hinge regions with limited structural flexibilities [35]. And 1HZH (IgG1) showed a crystallized antibody “with distorted arrangements of the Fab arms”, which was interpreted as a snapshot of an array of in-solution conformations, indicating structural flexibility [36]. Interestingly, not too long ago, an X-ray structure of a full-length IgG4 with a “distorted λ-shape” was published (6GFE). One of its Fab arms was strongly oriented towards the antibody’s Fc part [37]. But this structure was marked to stem from an antibody with “exceptional conformational diversity”. A putative explanation for the enhanced adoption of the “Y-shape” IgG conformation in crystals might be deduced from the observation of a self-assembly process of “Y-shaped” IgG, which happens when antibodies are deposited on smooth surfaces, e.g., for performing atomic force microscopy [38].

Methods which are capable of investigating higher-order protein structures, such as X-ray crystallography [39], NMR [40], and cryo-electron microscopy [41], typically provide information on static 3D structures, which may be regarded as snapshots of distinct protein conformations. Similarly, in-silico structure prediction tools, such as AlphaFold3 [42] and I-TASSER [43], also provide sets of high-confidence and, again, stagnant 3D protein structure models. The “frozen” 3D structures of macromolecules, such as antibodies obtained from these methods, leave very strong impressions on how biomolecules look, but not always do they provide enough information about how these might fulfill their dynamic in-vivo functions.

To close the gap between static 3D protein structures and their time-dependent behaviors in biological environments, future experimental studies on multi-domain protein structure conformations might include sophisticated computational approaches, such as homology modeling, fold recognition, and ab initio modeling [44,45,46]. And for further in-silico structure flexibility estimations of multi-domain proteins, the inclusion of molecular dynamics analyses, such as root mean square deviation or radii of gyration examination, should be beneficial as well [47,48]. Structural flexibility of IgG was in-silico modeled [49,50], and it has become clear that in-silico protein unfolding behavior replicates the behavior in solution and is in line with in-vacuo observations [51].

Further hints which point towards softening the general statement that antibodies (solely) assume “Y-shaped” conformations in solutions are provided by analyzing chemical cross-linking experiments with results suggesting that at least a temporary close vicinity of Fab and Fc elements was assumed in solutions, referred to as the “closed” conformation or “m-shape” [52,53]. While one could theoretically argue that cross-linking experiments could have caused artefactual proximities of Fab and Fc regions between neighboring IgG molecules, there are several other data that stand in line with the assumption that antibodies could adopt other than “Y-shape” in-solution conformations. Neutron spin echo spectroscopy showed remarkable flexibility in Fab-to-Fc orientations [54]. In addition, the dynamics of antibody structure changes in solution had been determined by studying single particles using cryo-electron tomography, and the results revealed great variability in the positioning of the Fab arms relative to the Fc stems of antibodies [55,56].

Our experimental ion mobility spectra and determined CCSs, as well as the computed CCSs from 3D protein structures, agree across charge states and over a large molecular mass range of compactly folded peptides, proteins, and protein complexes, and our results stand in agreement with previous observations [31,57]. The acceptance has grown that protein structures adopted in the condensed phase are largely maintained during desorption into the gas phase under “native” mass spectrometry conditions, making ion mobility mass spectrometry a prime method for analyzing higher-order protein structures and structural changes on a global scale. The theorem that the most compact protein structures correspond to the ion charge-state distributions with the fewest charges and, simultaneously, to ion series with the smallest CCSs is not questioned anymore. Likewise, assignments of charge-state envelopes with the highest charge states to denatured protein structures, e.g., from “non-native” solution conditions, were found self-explanatory when studying single-domain proteins [17,18]. Investigating shapeshifting multi-domain proteins by ESI ion mobility mass spectrometry presents a logical expansion of this protein analytical approach. But to assign more than one “native” in-solution structures to specific and distinct ion signal envelopes of the mass spectra requires additional information. The requested additional information can be obtained by ion mobility mass spectrometry and/or when there are 3D protein structures or structural models available. Computational data, particularly molecular dynamics calculations, can be added for clarification as well. Comparisons of computed CCSs with experimentally determined CCSs indeed facilitate the assignment of a given charge structure from the ESI mass spectrum to a corresponding 3D structure.

Shapeshifting characteristics were observed with protein G’e and all four investigated IgGs. According to our computations and our experimental data, the plus/minus 25+ charged IgG ion signals belong to the respective ion charge-state envelope from IgGs with “m-shaped” conformations according to our interpretation. In contrast to previous reports, we state that the in-solution “m-shape” IgG conformations postulated by us are mostly maintained in the gas phase, i.e., they follow the rule which was deduced from all other compactly folded proteins. The previously suggested process of collapsing, i.e., the rapid transition of (distorted) in-solution “Y-shape” conformations [58] as the cause of the “m-shape” IgG conformations in the gas phase [59,60], is not completely ruled out, but it is not regarded as the sole reason for the presence of “m-shape” IgG conformations in the gas phase. Note, regardless of their formation, the experimentally determined CCSs from the compact IgG ions with around 25+ charges are much too small when compared to CCSs computed from the “Y-shaped” 3D structures of IgGs [25,61,62].

As shown here, when capillary voltages are set very low, “native” ESI desolvation conditions can be achieved, by which IgG ions with higher charge states, centering on the 37+ charge state, are obtained in addition to the charge-state envelope which centers on the 25+ charge state. Intriguingly, the CCSs of these higher charge-state ions of the four investigated antibodies match those which are computed from the IgG X-ray structures where IgGs are Y-shaped. Thus, it is tempting to speculate that these higher charged ions of the four investigated antibodies had not collapsed during the ESI process but represented extended/folded “Y-shaped” IgG conformations which survived transition into the gas phase and stayed extended/folded, at least as long as it took these ions to travel through the mass spectrometer instrument.

Interestingly, in another study, a set of low-intensity ion signals in the *m*/*z* 3000−4500 region, referring to the same average mass of an IgG (148.7 kDa) but with a wider envelope of higher charge states than typically recorded, was characterized as “in-solution partially unfolded antibody species” present in the mass spectra at trace levels [63]. Because of the specific nature of their observation, the authors argued against a purely method-induced artifact, but they did not attempt to assign specific IgG in-solution conformations to the ions from this additional ion series. Also in line with our assignments of the four investigated multiply protonated IgG ions to represent either compact “m-shape” or extended “Y-shape” conformations are “soft-landing” data, which, upon ESI desorption, showed the predominant compacted/folded conformations of antibodies after their travel through the vacuum of a mass spectrometer [64]. More importantly, in these “soft landing” experiments, smaller fractions of non-collapsed IgGs, i.e., with extended/folded “Y-shape” conformations, were recorded as well [65]. Moreover, the assumption that “native” mass spectrometry enables the distinction of “native” IgG conformations in the gas phase which reflect the in-solution situation, as reported here, stands in agreement with analytical ultracentrifugation studies where X-ray and neutron distance distributions confirmed the simultaneous existence of two stable IgG conformations in buffered solutions [66].

Our study’s result interpretations concerning multi-domain proteins are limited by the fact that we base their extended/folded in-solution structure assignments on gas-phase CCS values of protein G’e conformers and of four antibodies from which the extended/folded “native” IgG conformation—Y-shaped IgG according to our interpretation—had been determined experimentally. To our knowledge, the number of reports which describe the existence of higher charge states centering on the 38+ charge state in “native” IgG preparations, i.e., solutions of IgG dissolved in volatile buffers with neutral pH, remains scarce. By contrast, the number of reports on denatured IgG with broad distributions of high charge states, centering on 50+ ion signals or higher, as well as the compacted/folded “native” IgG structure with ion signals centering on the 25+ charge state under “native” conditions—m-shaped IgG according to our interpretation—is substantial. But when associated with structure, the respective ions of the charge envelope with a maximum at 25+ either have been erroneously assigned as being derived from Y-shaped IgG conformers or, more recently, from gas-phase-collapsed m-shaped IgGs. Moreover, the existence of in-solution equilibria of multi-domain protein conformers was deduced by us based on general chemical principles instead of direct molecular dynamics investigations. Likewise, we have not studied protein structure dependencies, or, more precisely, multi-domain protein conformer dependencies of solvent compositions, which may shift the balance of an equilibrium [67]. In summary, the interpretation of our results is based on a total of fifty wet-lab datasets from five peptides, seven single-domain proteins, three protein complexes, and five multi-domain proteins, i.e., protein G’e and four IgGs (Tables S4, S8, S9 and S11–S13). We added matching dry-lab data (Tables S5 and S10) and compared our results with published data (Tables S4, S8 and S11).

We conclude that the presence of different in-solution conformations of multi-domain proteins is readily and reliably revealed by nanoESI ion mobility mass spectrometry. We infer that the four investigated IgGs can adopt more than one conformation in solution and that conformation equilibria may likely shift upon experiencing external influences. Shifting the antibody conformations upon antigen binding is suggested to take place by the conformational rearrangement hypothesis, where subsequent binding of an immune complex by the immune cells’ Fc receptors then triggers opsonophagocytosis and/or activates further immune cell response mechanisms [1].

Despite the fact that CCS values are experimentally determined in the gas phase, we encourage future careful (re-) examination of the gas-phase collision-induced unfolding (CIU) of multi-domain proteins in general and of IgGs in particular [68,69], which may turn out to be fruitful for better understanding in-solution conformational changes in multi-domain proteins. And in the context of biological function and/or cellular signaling, conformation studies of soluble IgG-containing immune complexes [70] using “native” mass spectrometry, including CCS determinations, should be of high interest as well.

## 4. Materials and Methods

### 4.1. Peptides, Proteins, and Complexes

Angiotensin II (Homo sapiens) (1) [71] was purchased from Merck (Sigma-Aldrich), Taufkirchen, Germany (ordering no. A9650, Lot no. 058K5104). Bradykinin (Homo sapiens) (2) [72] was purchased from Bachem, Bubendorf, Switzerland (ordering no. H-1970). Angiotensin I (Homo sapiens) (3) [73] was purchased from Merck (Sigma-Aldrich), Taufkirchen, Germany (ordering no. A9650, Lot no. 058K5104). Melittin/P01501 (Apis mellifera) (4) [74] was purchased from Bachem, Bubendorf, Switzerland (ordering no. 4030808, Lot no. 1000029758). Insulin/P01317 (Bos taurus) (5) [71] was purchased from Merck (Sigma-Aldrich), Taufkirchen, Germany (ordering no. I5500). Ubiquitin/P0CG48 (Bos taurus) (6) [75] was purchased from Merck (Sigma-Aldrich), Taufkirchen, Germany (ordering no. U6253, Lot no. 000423189, source SLCP6637). Cytochrome C/P00004 (Equus caballus) (7) [76] was purchased from Merck (Sigma-Aldrich), Taufkirchen, Germany (ordering no. C2506, Lot no. 017K7004). Holo myoglobin/P68082 (Equus caballus) (8) [77] was purchased from Merck (Sigma-Aldrich), Taufkirchen, Germany (ordering no. M1882, Lot no. 60K7007). Pepsin/P00791 (Sus scrofa) (9) [78] was purchased from Merck (Sigma-Aldrich), Taufkirchen, Germany (ordering no. P6887). Ovalbumin/P01012 (Gallus gallus) (10) [79] was purchased from Merck (Sigma-Aldrich), Taufkirchen, Germany (ordering no. A7641, Lot no. SLBL9222V). IgG-Fc/P01857 (Homo sapiens) (11) [80,81] was purchased from Abcam, Cambridge, UK (ordering no. ab90285, Lot no. GR3266121-1). Streptavidin/P22629 (Streptomyces avidinii) (12) [82] was purchased from Carl Roth GmbH & Co KG, Karlsruhe, Germany. Hemoglobin/P69905 and P68871 (Homo sapiens) (13) [83] was purchased from Merck (Sigma-Aldrich), Taufkirchen, Germany (ordering no. H7379, Lot no. 39H7605). Serum albumin/P02769 (Bos taurus) (14) [77] was purchased from Merck (Sigma-Aldrich), Taufkirchen, Germany (ordering no. A4378, Lot no. 16H937). Alcohol dehydrogenase/P00330 (Saccharomyces cerevisiae) (15) [84] was purchased from Merck (Sigma-Aldrich), Taufkirchen, Germany (ordering no. A7011, Lot no. 021K7405). Protein G’e/Q54181 (A1) [85] was purchased from Merck (Sigma-Aldrich), Taufkirchen, Germany (ordering no. P4689, Lot no. SLBX4122). HAM1101 (A2α) was a gift from AdrenoMed AG, Henningsdorf, Germany. αMSP1_19_ (A2β) [86] (G17.12) (ordering no. PABW-101, Lot no. 2509FY02) was purchased from Creative Biolabs, Shirley, NY, USA. αHis-tag antibody (A2γ) [87] (mouse monoclonal, clone AD1.1.10) (ordering no. MCA 1396) was purchased from Bio-Rad Laboratories Inc., Hercules, CA, USA. Rituximab (A2δ) [88] (Batch no. H0013) was produced by Roche Ltd., Welwyn Garden City, UK.

### 4.2. Preparation of Stock Solutions

Stock solutions of 0.09 mg/mL peptide to 3.66 mg/mL protein/complex (µM solutions) were prepared by dissolving lyophilized powders in 200 mM ammonium acetate, pH 6.9. Pepsin was dissolved in 2% acetic acid/methanol (95:5, *v*/*v*). Human IgG-Fc was purchased as a solution in 0.79% Tris HCl buffer containing 1.16% sodium chloride and 0.05% sodium azide. Aliquots of 25–100 µL from solutions that contained holo-myoglobin, human IgG-Fc, streptavidin, hemoglobin, and alcohol dehydrogenase were rebuffered into 200 mM ammonium acetate, pH 6.9, using centrifugal filters (Merck Millipore, Carrigtwohill, Ireland) with 3 kDa, 30 kDa, or 50 kDa cutoff, respectively. Prior to buffer exchange, the Rituximab stock solution was diluted 1:5 by mixing 200 µL of Rituximab stock solution with 800 µL of 200 mM ammonium acetate buffer, pH 6.9. For buffer exchange by ultrafiltration, volumes of 50–200 µL of HAM1101 stock solution (A2α), αMSP1_19_ stock solution (A2β), αHis-tag antibody stock solution (A2γ), and diluted Rituximab (A2δ) were rebuffered into 200 mM ammonium acetate buffers, pH 6.9, by transferring the antibody solutions onto one centrifugal filter unit, each with a molecular mass cut-off of 50 kDa (Merck Millipore, Carrigtwohill, Ireland), together with 300–450 µL of 200 mM ammonium acetate buffer, pH 6.9. These solutions were each centrifuged for 10 min at room temperature at 13,400 rpm. The filtrates were discarded, and 400 µL of the 200 mM ammonium acetate buffer were added to the retentates. Centrifugation, discarding of filtrates, and refilling procedures were repeated eight times. Then, the filters were inverted, placed into new vials, and centrifuged for 5 min at 4500 rpm at room temperature to concentrate the volumes of the retentates to approximately 20–40 µL (antibody stock solutions) [89].

### 4.3. Peptide and Protein Concentration Determinations

Peptide and protein concentrations of stock solutions and antibody working solutions were determined using the Qubit^®^ Protein Assay Kit (Life Technologies Corp., Eugene, OR, USA), as published in [82]. From each peptide/protein stock solution, a volume of 1–10 µL was mixed with 190–199 µL of the Qubit^®^ working solution. After incubation periods of 15 min, protein concentrations were read using a Qubit^®^ 2.0 Fluorimeter (Invitrogen AG, Carlsbad, CA, USA).

### 4.4. Preparation of Nanospray Emitters

NanoESI emitters for offline measurements were prepared in-house as previously described [86]. Briefly, borosilicate glass tubes (BF 100-50-10) with 0.5 mm inner and 1 mm outer diameters were used to produce emitters using a P-1000 Flaming/Brown Micropipette Puller System (Sutter Instrument, Novato, CA, USA). Emitters were gold-coated under an argon atmosphere with an SCD005 Sputter Coater (BALTEC AG, Balzers, Liechtenstein) by setting the following parameters: current of 20 mA, sputter time duration of 180 s, 5 cm working distance between the emitters and the gold foil target, and argon gas pressure of 0.5 mbar.

### 4.5. Preparation of Working Solutions and Loading of Nanospray Emitters

Prior to nanoelectrospray mass spectrometry, aliquots of the appropriate peptide/protein/complex working solutions were prepared by diluting stock solutions with 200 mM ammonium acetate, pH 6.9, to yield final peptide/protein/complex concentrations of 9 ng/µL up to 0.65 µg/µL. For offline nanoESI measurements, a volume of 3 μL of the respective peptide/protein/complex working solution was loaded into a gold-coated nanoESI emitter using a microloader pipette tip (Eppendorf, Hamburg, Germany).

### 4.6. 3D Structures

The PepFold4 de novo peptide structure prediction server (https://mobyle2.rpbs.univ-paris-diderot.fr/cgi-bin/portal.py#forms::PEP-FOLD4 (accessed on 30 December 2025)) was used for modeling 3D structures of angiotensin II, bradykinin, and angiotensin I [90]. The I-TASSER version 4.4 protein structure prediction server (https://aideepmed.com/I-TASSER/ (accessed on 2 July 2025)) was applied to model the compacted protein G’e structure model [91]. The extended protein G’e structure model was downloaded from the Uniprot database (entry Q54181; https://alphafold.ebi.ac.uk/entry/Q54181 (accessed on 30 December 2025)). The published protein 3D coordinates of serum albumin (3v03.pdb), streptavidin (1swb.pdb), IgG-Fc (1fc1.pdb), ovalbumin (1ova.pdb), pepsin (4pep.pdb), holo myoglobin (1mbn.pdb), cytochrome c (1hrc.pdb), ubiquitin (1ubq.pdb), insulin (6qq7.pdb), and melittin (2mlt.pdb) were downloaded from the RCSB Protein Data Bank (https://www.rcsb.org/). 3D structures were visualized using UCSF ChimeraX v1.8 molecular visualization software [92,93]. For molecular modeling, only the amino acid sequence was required for the task.

### 4.7. CCS Calculations

CCSs were determined using the 3D coordinates and Collidoscope software, which is a trajectory method for collisional cross-section modeling (version coll_8_1_19.tar.gz) [27]. The program was run on a computer using default parameters: temperature, 298.15 K; minimum energy, 0.5 RT; maximum energy, 8 RT; energy states, 10; particle type, spherical; integration method, Euler; trajectory setting, none; carrier gas, He. The average CCS was determined using average cross-section values from ten energy state levels.

### 4.8. Offline nanoESI-MS

Mass spectra were acquired on a Synapt G2-S instrument (Waters MS-Technologies, Wilmslow, UK). Instrument calibration was performed using a sodium iodide solution with a concentration of 2 mg/mL, which was dissolved in isopropanol/water (50:50, *v*/*v*). All mass spectra were acquired in positive-ion mode, applying a mass window from *m*/*z* 400 to *m*/*z* 10,000 in duplicate. NanoESI measurements were performed with the following instrument settings: capillary voltage: 0.85–1.8 kV; sample cone voltage: 10 V (serum albumin: 120 V); source offset voltage: 10 V (serum albumin: 120 V); source temperature: 40 °C; cone gas flow: 9 L/h; purge gas flow: 27 mL/h; nano-flow gas pressure: 0.21 bar; trap collision voltage: 2.0 V; transfer collision voltage: 0 V; trap gas flow: 3.0 mL/min. Data acquisition and processing were done with MassLynx software version 4.1 (Waters MS-Technologies, Wilmslow, UK) [89,94].

### 4.9. Ion Mobility Analyses

Ion mobility measurements were performed using a Synapt G2-S instrument (Waters Corporation, Wilmslow, UK) equipped with a travelling wave ion mobility (TWIM) cell. Nitrogen was the neutral buffer gas for all TWIM experiments [95]. All mass spectra were acquired in positive-ion mode, applying a mass window from *m*/*z* 400 to *m*/*z* 10,000 in duplicate. Instrument settings were as follows: capillary voltage: 0.85–1.8 kV; sample cone voltage: 10 V (serum albumin: 120 V); source offset voltage: 10 V (serum albumin: 120 V); source temperature: 40 °C; cone gas flow: 9 L/h; purge gas flow: 27 mL/h; nano-flow gas pressure: 0.21 bar; trap collision voltage: 2.0 V; transfer collision voltage: 0 V; trap gas flow: 5.0 mL/min; helium cell gas flow: 140 mL/min; IMS gas flow: 50.0 mL/min; IMS wave velocity: 1600 m/s; wave height voltage: 30.0 V. Data acquisition, arrival time determinations, and processing were done with MassLynx software version 4.1 (Waters MS-Technologies, Wilmslow, UK [95]. TWIM calibration was done using alcohol dehydrogenase, bovine serum albumin, and myoglobin for the mass range above 20 kDa and cytochrome c, ubiquitin, and angiotensin II for the mass range below 20 kDa, using published CCS values [30,96,97,98,99,100] and applying the published algorithms for logarithmic fits [96,101] and linear fits [102] via Excel spreadsheet-based calculations. All mass spectra recorded in duplicate were subjected to data analyses, including averaging and calculation of standard deviations.

## Figures and Tables

**Table 1:** Collisional cross-sections of protein G’e (A1) conformers.

Mode ^(a)^	A1I ^(b)^		A1II ^(b)^		A1III ^(b,c)^	
	z	CCS [Å^2^]	z	CCS [Å^2^]	z	CCS [Å^2^]
exp.	9	2260 ± 2	14	3404 ± 0	24	5742 ± 0
calcd	9	2673	14	3969	n.a.	n.a.

^(a)^ Determination mode; exp.: experimentally by ion mobility mass spectrometry (n = 2). calcd: calculated with Collidoscope. ^(b)^ Conformers: I: compact; II: extended; III: denatured. ^(c)^ n.a.: not applicable.

**Table 2:** Collisional cross-sections of IgG immunoglobulin (A2) conformers.

Analyte (A2)	Example (x) ^(a)^	Mode ^(b)^	A2xI ^(c)^		A2xII ^(c)^		A2xIII ^(a,c)^	
			z	CCS [Å^2^]	z	CCS [Å^2^]	z	CCS [Å^2^]
HAM1101	α	exp.	25	7605 ± 0	37	10,127 ± 18	45	11,903 ± 223
αMSP1_19_	β	exp.	25	7494 ± 12	38	10,152 ± 18	45	11,731 ± 23
αHis-tag	γ	exp.	25	7519 ± 1	37	10,089 ± 35	44	11,437 ± 64
Rituximab	δ	exp.	25	7519 ± 0	37	10,087 ± 70	47	12,477 ± 206
IgG	n.a.	calcd	25	7291	37	9948	n.a.	n.a.

^(a)^ n.a.: not applicable. ^(b)^ Determination mode; exp.: experimentally by ion mobility mass spectrometry (n = 2). calcd: calculated with Collidoscope. ^(c)^ Conformations: I: compact; II: extended; III: denatured.

## Data Availability

The mass spectrometry data presented in this study have been deposited in the PRIDE partner repository of the ProteomeXchange Consortium under the dataset identifier PXD076256.

## Supplementary Materials

The following supporting information can be downloaded at https://www.mdpi.com/article/10.3390/ijms27177662/s1.

## Abbreviations

The following abbreviations are used in this manuscript:

CCS	Collisional cross-section
ESI	Electrospray ionization
ITEM	Intact Transition Epitope Mapping
ITEM-ONE	ITEM—One-step Non-Covalent Force Exploitation
ITEM-TWO	ITEM—Thermodynamic Weak-Force Order
ITEM-THREE	ITEM—Targeted High-Energy Rupture of Extracted Epitopes
ITEM-FOUR	ITEM—Force differences between Original and Unusual Residues
ITEM-FIVE	ITEM—Force Interferences by Variable Extensions
ITEM-SIX	ITEM—Serological Inspection by Epitope Extraction
*m/z*	Mass-to-charge ratio
PDB	Protein database
PRIDE	Proteomics identifications database
Q-ToF	Quadrupole time of flight
TIC	Total ion current
TWIM	Traveling wave ion mobility
