# Supplementary material for: Four IgG Antibodies and Protein G Are Shapeshifters
Source: Int J Mol Sci. 2026 Aug 26;27(17):7662. doi: 10.3390/ijms27177662 (PMC13566121; doi:10.3390/ijms27177662)
Supplement: Supplementary file 1 [file ijms-27-07662-s001.zip › ijms-4461245-supplementary.pdf]

Four IgG Antibodies and Protein G Are Shapeshifters

Michael O. Glocker [1,*], Manuela Ruß [1], Cornelia Koy [1], Michael Kreutzer [1], Fiona T. I. Melder [1], Yelena Diebler [1], Harald Illges [2] and Kwabena F. M. Opuni [3,4]

[1] Proteome Center Rostock, Medical Faculty and Natural Science Faculty, University of Rostock, Schillingallee 69, 18057 Rostock, Germany
[2] Department of Applied Natural Sciences, Immunology and Cell Biology, Institute of Functional Gene Analytics, University of Applied Sciences Bonn-Rhein-Sieg, von-Liebig-Str. 20, 53359 Rheinbach, Germany; harald.illges@h-brs.de
[3] Department of Pharmaceutical Chemistry, School of Pharmacy, College of Health Science, University of Ghana, Legon P.O. Box LG43, Ghana; kfopuni@ug.edu.gh
[4] Food and Drugs Authority Ghana, Cantonments, Accra P.O. Box CT2783, Ghana
* Correspondence: michael.glocker@uni-rostock.de; Tel.: +49-381-494-4930; Fax: +49-381-494-4932

Supplemental Figures 2

Supplemental Tables 9

Supplemental Figures

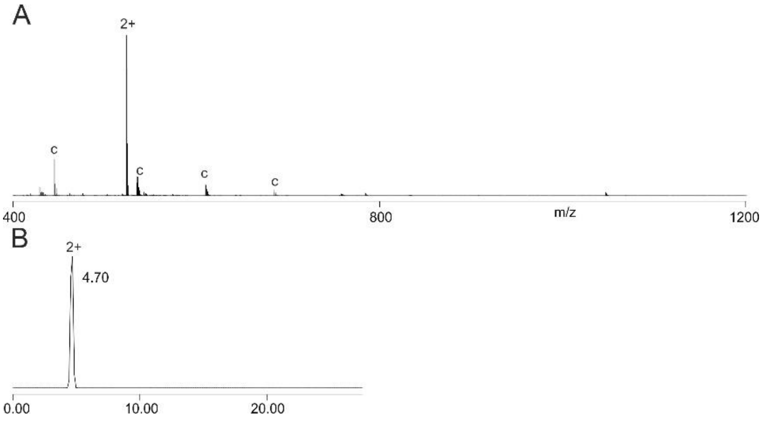

Figure S1: NanoESI mass spectrum of angiotensin II (homo sapiens) and drift time chromatogram (1). **A:** Doubly charged ion signal. Charge state is given. Ion signals labeled with c are from poly-siloxane contaminants. **B:** Drift time chromatogram. Retention time and charge state is indicated. Solvent: 200 mM ammonium acetate, pH 6.9.

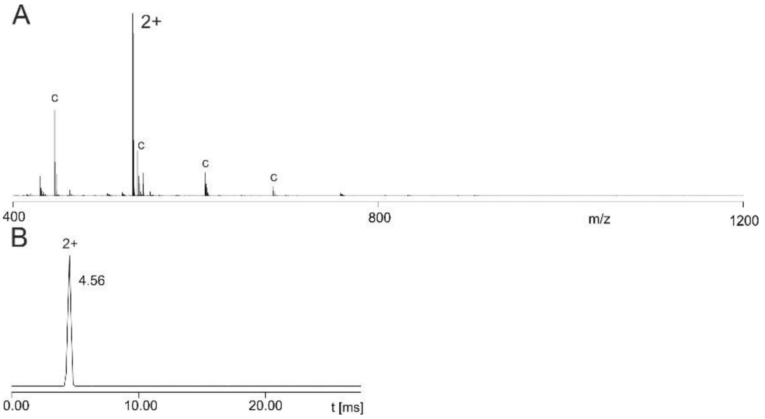

Figure S2: NanoESI mass spectrum of bradykinin (homo sapiens) and drift time chromatogram (2). **A:** Doubly charged ion signal. Charge state is given. Ion signals labeled with c are from poly-siloxane contaminants. **B:** Drift time chromatogram. Retention time and charge state is indicated. Solvent: 200 mM ammonium acetate, pH 6.9.

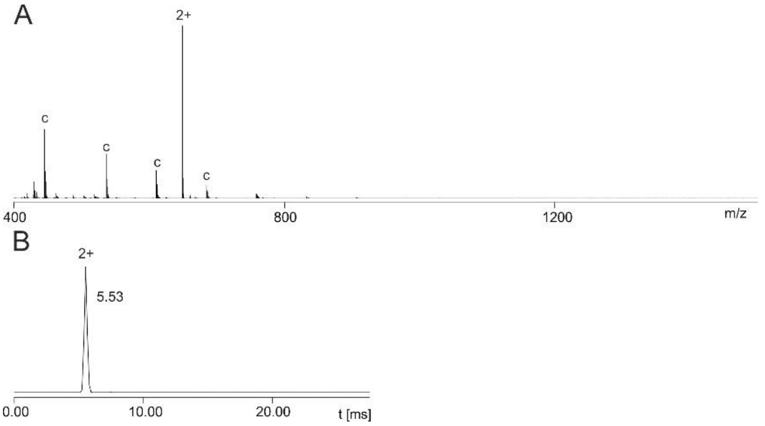

Figure S3: NanoESI mass spectrum of angiotensin I (homo sapiens) and drift time chromatogram (3). **A:** Doubly charged ion signal. Charge state is given. Ion signals labeled with c are from poly-siloxane contaminants. **B:** Drift time chromatogram. Retention time and charge state is indicated. Solvent: 200 mM ammonium acetate, pH 6.9.

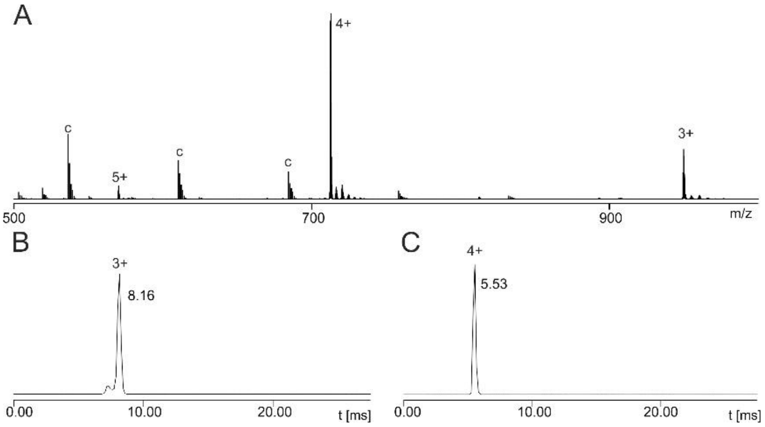

Figure S4: NanoESI mass spectrum of mellitin (apis mellifera) and drift time chromatograms of selected ions (4).
A: Multiply charged ion signals. Charge states are given. Ion signals labeled with c are from poly-siloxane contaminants. **B and C:** Drift time chromatograms. Retention times of individual ions and charge states are indicated. Solvent: 200 mM ammonium acetate, pH 6.9.

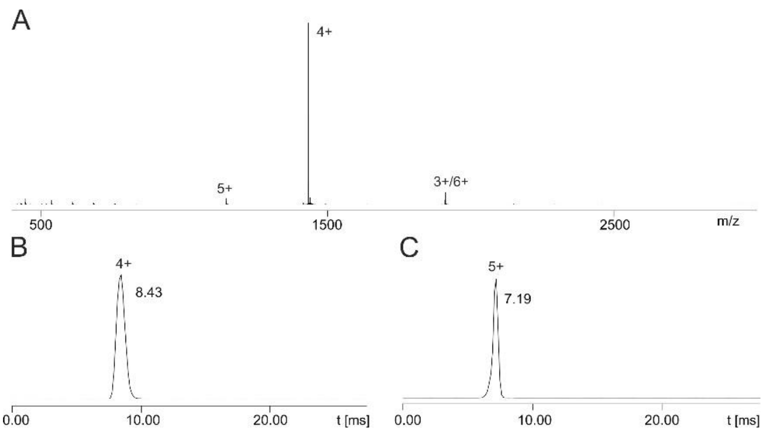

Figure S5: NanoESI mass spectrum of insulin (bos taurus) and drift time chromatograms of selected ions (5). **A:** Multiply charged ion signals. Charge states are given. **B and C:** Drift time chromatograms. Retention times of individual ions and charge states are indicated. Solvent: 200 mM ammonium acetate, pH 6.9.

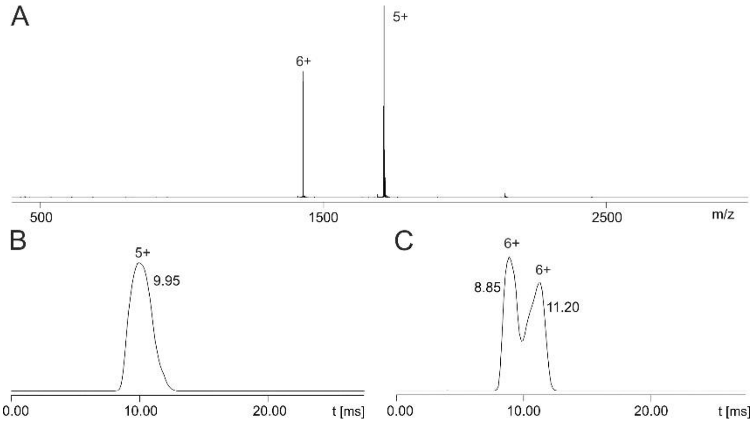

Figure S6: NanoESI mass spectrum of ubiquitin (bos taurus) and drift time chromatograms of selected ions (6). **A:** Multiply charged ion signals. Charge states are given. **B and C:** Drift time chromatograms. Retention times of individual ions and charge states are indicated. Solvent: 200 mM ammonium acetate, pH 6.9.

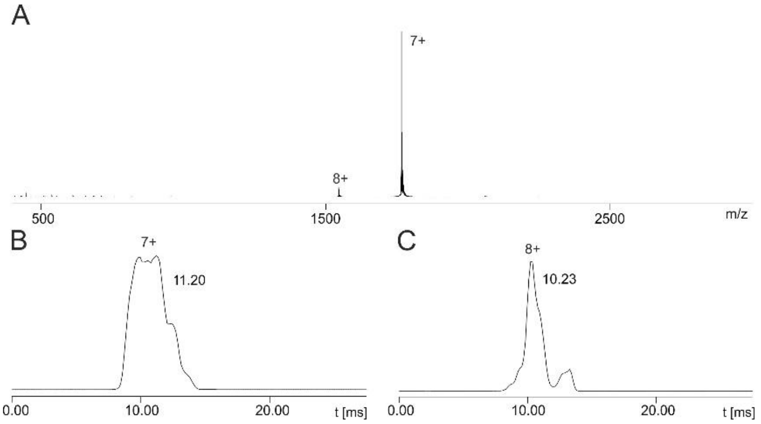

Figure S7: NanoESI mass spectrum of cytochrome C (equus caballus) and drift time chromatograms of selected ions (7). **A:** Multiply charged ion signals. Charge states are given. **B and C:** Drift time chromatograms. Retention times of individual ions and charge states are indicated. Solvent: 200 mM ammonium acetate, pH 6.9.

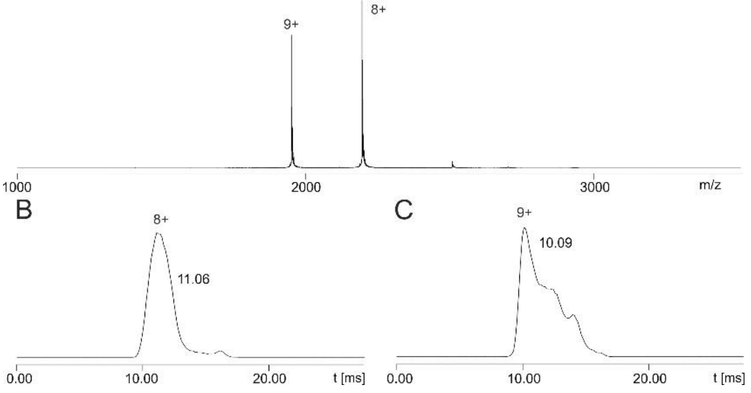

Figure S8: NanoESI mass spectrum of holo-myoglobin (equus caballus) and drift time chromatograms of selected ions (8). **A:** Multiply charged ion signals. Charge states are given. **B and C:** Drift time chromatograms. Retention times of individual ions and charge states are indicated. Solvent: 200 mM ammonium acetate, pH 6.9.

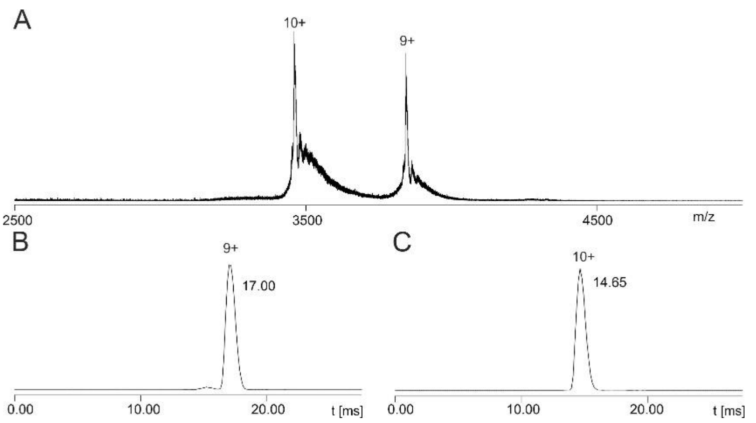

Figure S9: NanoESI mass spectrum of pepsin (sus scrofa) and drift time chromatograms of selected ions (9).
A: Multiply charged ion signals. Charge states are given. **B and C:** Drift time chromatograms. Retention times of individual ions and charge states are indicated. Solvent: 2% acetic acid/methanol (95/5, v/v), pH 2.

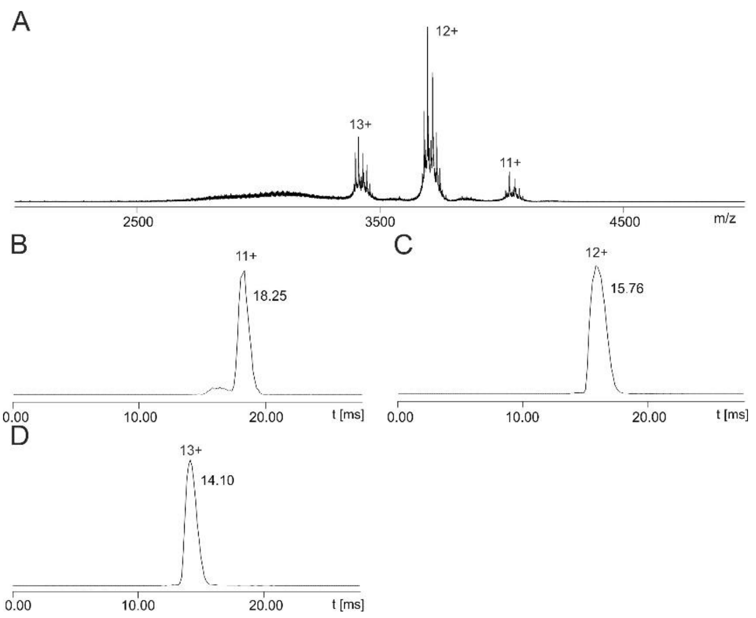

Figure S10: NanoESI mass spectrum of ovalbumin (gallus gallus) and drift time chromatograms of selected ions (10).
A: Multiply charged ion signals. Charge states are given. **B - D:** Drift time chromatograms. Retention times of individual ions and charge states are indicated. Solvent: 200 mM ammonium acetate, pH 6.9.

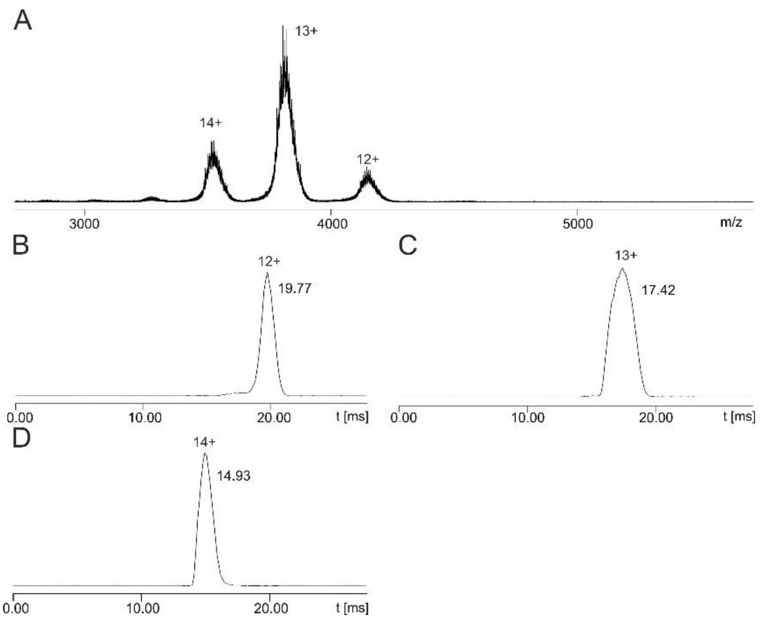

Figure S11: NanoESI mass spectrum of IgG- Fc (homo sapiens) and drift time chromatograms of selected ions (11).
A: Multiply charged ion signals. Charge states are given. **B - D:** Drift time chromatograms. Retention times of individual ions and charge states are indicated. Solvent: 200 mM ammonium acetate, pH 6.9.

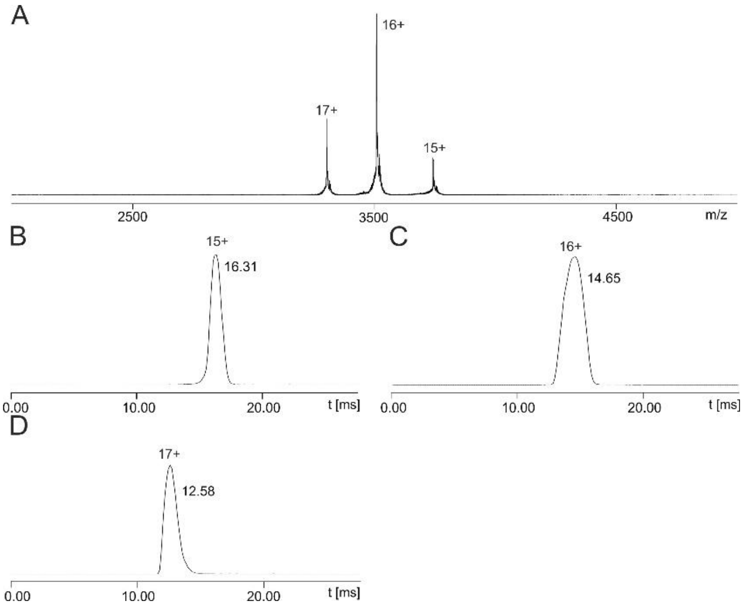

Figure S12: NanoESI mass spectrum of streptavidin (streptomyces avidinii) and drift time chromatograms of selected ions (12).
A: Multiply charged ion signals. Charge states are given. **B - D:** Drift time chromatograms. Retention times of individual ions and charge states are indicated. Solvent: 200 mM ammonium acetate, pH 6.9.

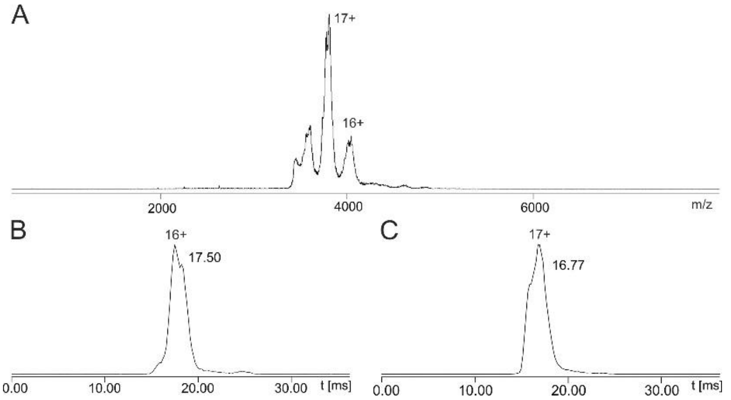

Figure S13: NanoESI mass spectrum of hemoglobin (homo sapiens) and drift time chromatograms of selected ions (13).
A: Multiply charged ion signals. Charge states are given. **B and C:** Drift time chromatograms. Retention times of individual ions and charge states are indicated. Solvent: 200 mM ammonium acetate, pH 6.9.

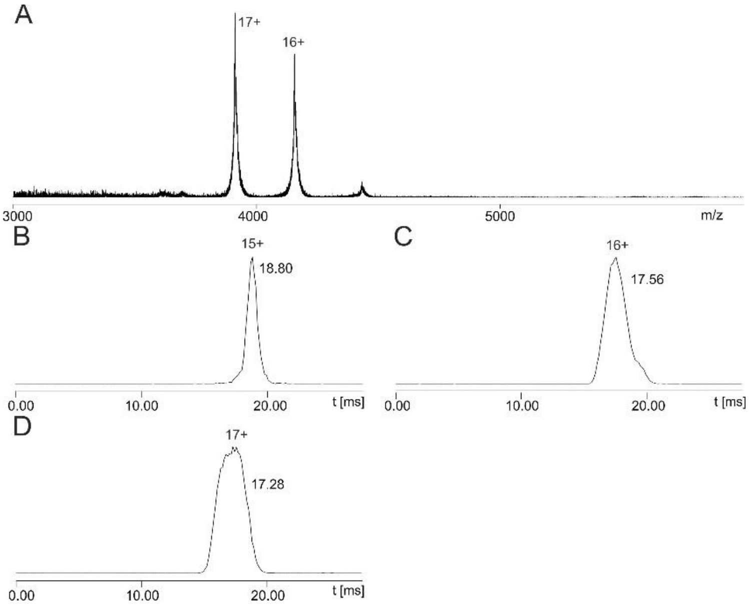

Figure S14: NanoESI mass spectrum of serum albumin (bos taurus) and drift time chromatograms of selected ions (14).
A: Multiply charged ion signals. Charge states are given. **B - D:** Drift time chromatograms. Retention times of individual ions and charge states are indicated. Solvent: 200 mM ammonium acetate, pH 6.9.

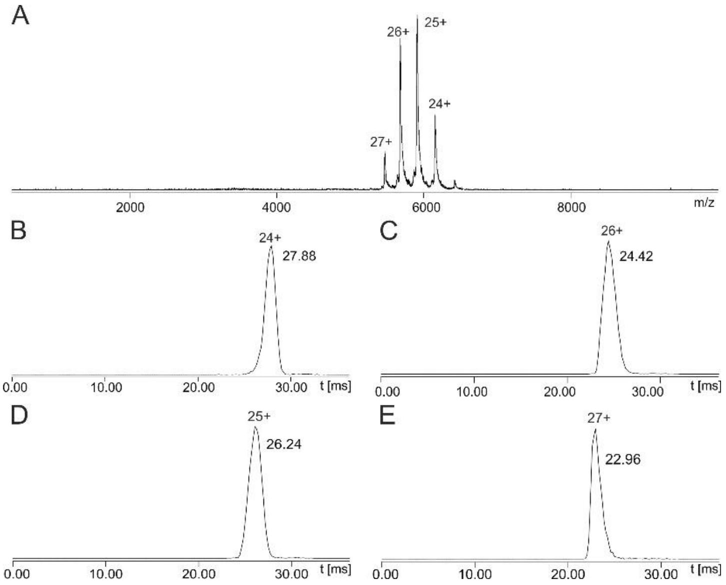

Figure S15: NanoESI mass spectrum of alcohol dehydrogenase (Saccharomyces cerevisiae) and drift time chromatograms of selected ions (15).
A: Multiply charged ion signals. Charge states are given. **B - E:** Drift time chromatograms. Retention times of individual ions and charge states are indicated. Solvent: 200 mM ammonium acetate, pH 6.9.

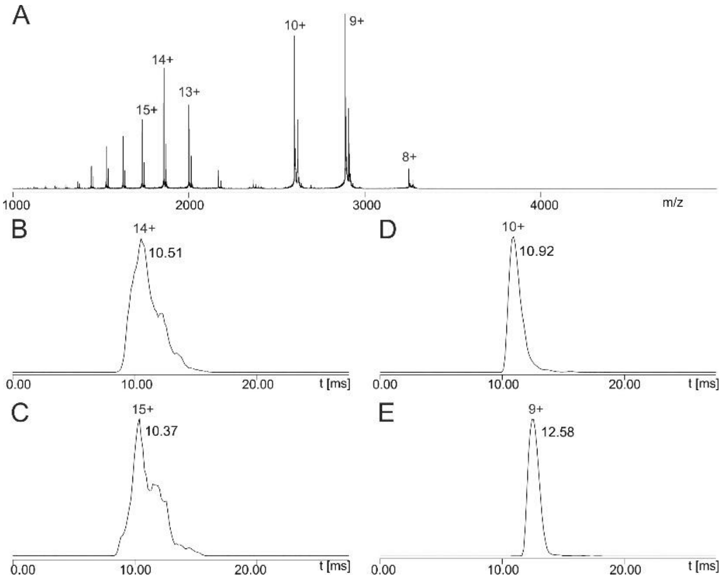

Figure S16: NanoESI mass spectrum of protein G'e (streptococcus sp.) and drift time chromatograms of selected ions (A1).
A: Multiply charged ion signals. Charge states are given. **B - E:** Drift time chromatograms. Retention times of individual ions and charge states are indicated. Solvent: 200 mM ammonium acetate, pH 6.9.

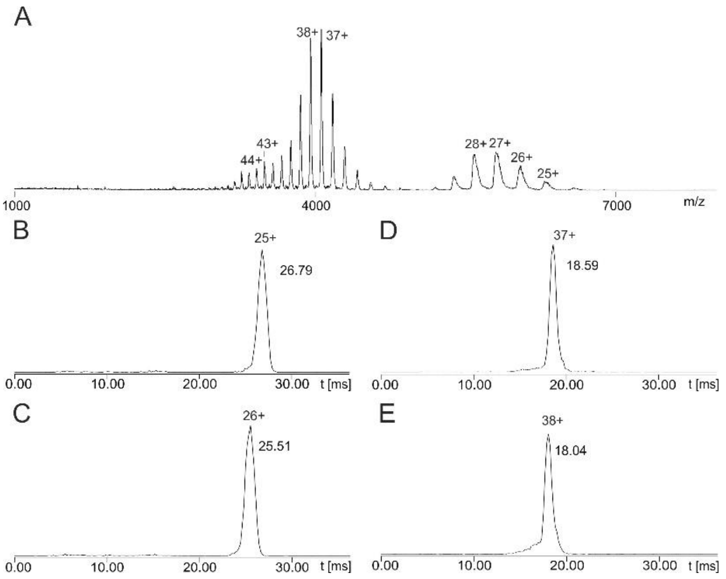

Figure S17: NanoESI mass spectrum of HAM1101 antibody and drift time chromatograms of selected ions (A2α).
A: Multiply charged ion signals. Charge states are given. **B - E:** Drift time chromatograms. Retention times of individual ions and charge states are indicated. Solvent: 200 mM ammonium acetate, pH 6.9.

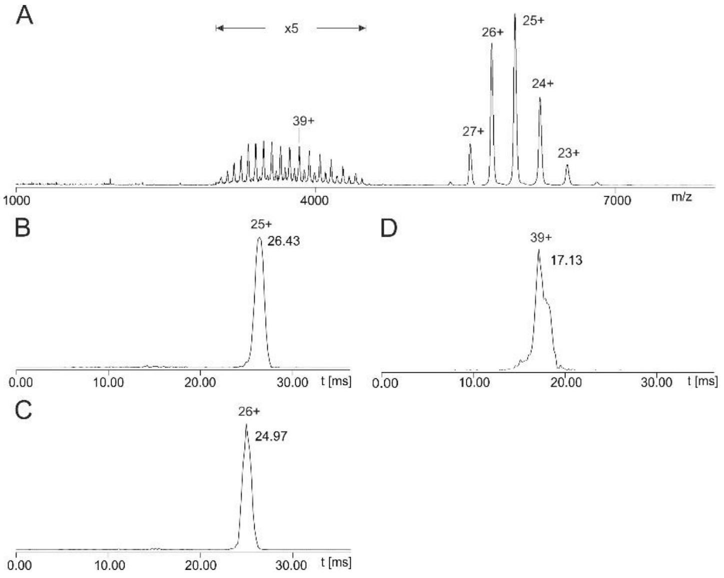

Figure S18: NanoESI mass spectrum of antiMSP1$_{19}$ antibody and drift time chromatograms of selected ions (A2β). **A:** Multiply charged ion signals. Charge states are given. **B - D:** Drift time chromatograms. Retention times of individual ions and charge states are indicated. Solvent: 200 mM ammonium acetate, pH 6.9.

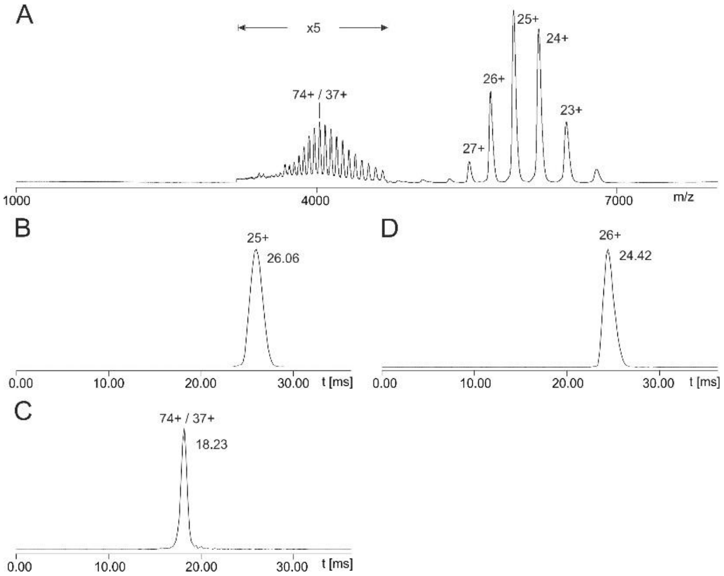

Figure S19: NanoESI mass spectrum of antiHis-tag antibody and drift time chromatograms of selected ions (A2γ). **A:** Multiply charged ion signals. Charge states are given. **B - D:** Drift time chromatograms. Retention times of individual ions and charge states are indicated. Solvent: 200 mM ammonium acetate, pH 6.9.

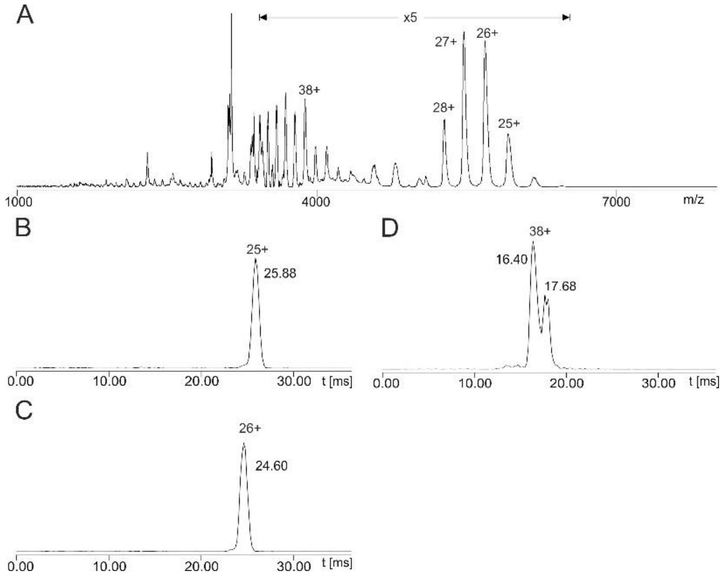

Figure S20: NanoESI mass spectrum of Rituximab antibody and drift time chromatograms of selected ions (A2δ). **A:** Multiply charged ion signals. Charge states are given. **B - D:** Drift time chromatograms. Retention times of individual ions and charge states are indicated. Solvent: 200 mM ammonium acetate, pH 6.9.

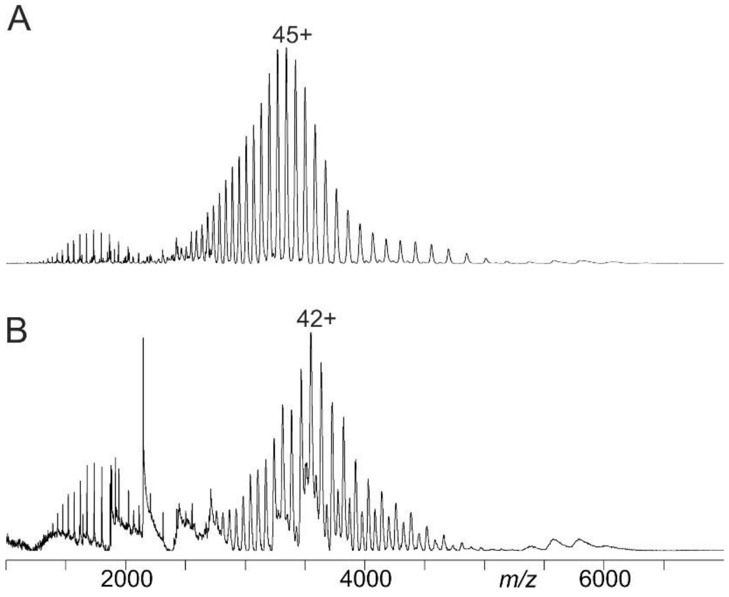

Figure S21: NanoESI mass spectra of antibodies at acidic pH.
A: HAM1101 (A2α). **B:** antiHis-tag antibody (A2γ). Charge states are indicated and CCS values are given. Solvent: 10 % acetic acid / MeOH (9:1, v/v), pH 3.

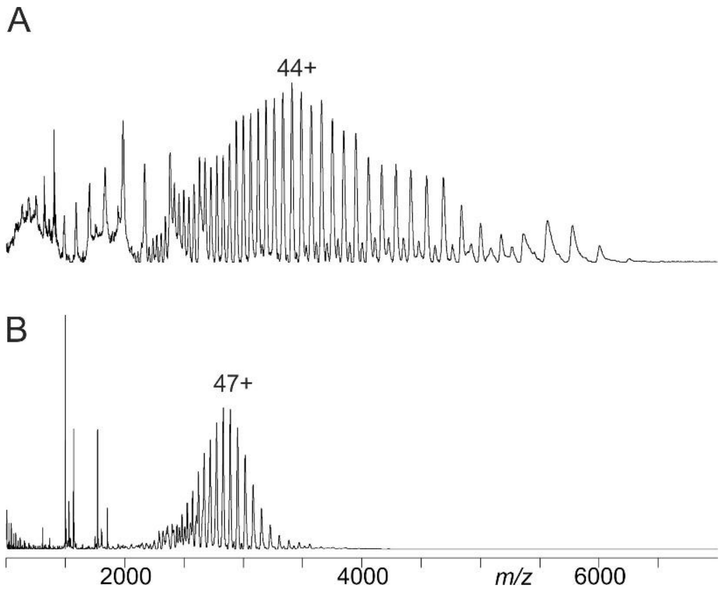

Figure S22: NanoESI mass spectra of antibodies at acidic pH.
A: antiMSP1$_{19}$ antibody (A2β). **B:** Rituximab (A2δ). Charge states are indicated and CCS values are given. Solvent: 10 % acetic acid / MeOH (9:1, v/v), pH 3.

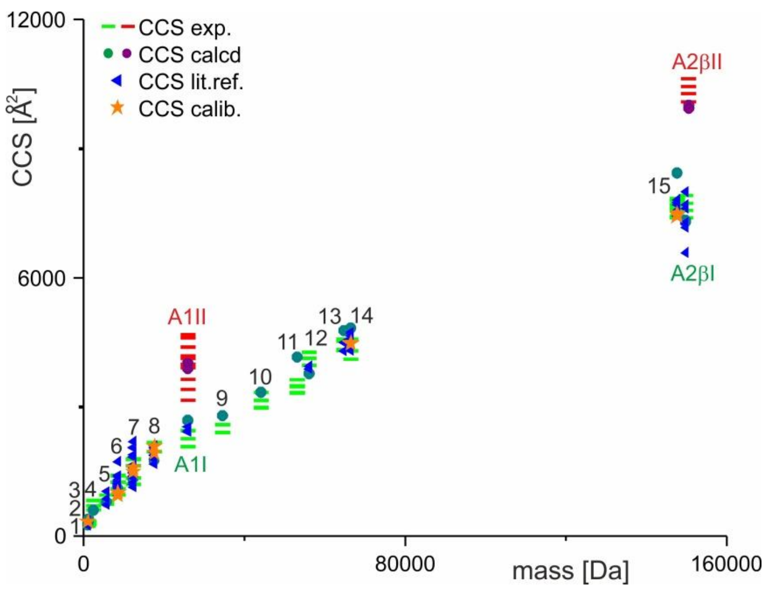

Figure S23: Collisional cross section over molecular mass of peptides, proteins, and complexes. 1: Angiotensin II. 2: Bradykinin. 3: Angiotensin I. 4: Melittin. 5: Insulin. 6: Ubiquitin. 7: Cytochrome C. 8: Myoglobin. 9: Pepsin. 10: Ovalbumin. 11: IgG Fc. 12: Streptavidin. 13: Hemoglobin. 14: Serum albumin. 15: Alcohol dehydrogenase. A1: Protein G'e. A2β: αMSP1$_{19}$. Horizontal lines show experimental values for different charge states recorded at pH 7. Filled circles represent computed values based on 3D structures or 3D structure models. Asterisks indicate values which were used for calibration. Green: compacted conformations (I). Red: extended conformations (II). For individual values see Supplemental Tables.

Supplemental Tables

Table S1: Amino acid sequences of peptides, single domain proteins and complexes.

no.	name / code (organism) [a)]	amino acid sequence [b)]	lit. ref
1	angiotensin II / n.a. (Homo sapiens)	DRVYIHPF	71
2	bradykinin / n.a. (Homo sapiens)	RPPGFSPFR	72
3	angiotensin I / n.a. (Homo sapiens)	DRVYIHPFHL	73
4	melittin / P01501 (Apis mellifera)	GIGAVLKVLTTGLPALISWIKRKRQQ	74
5	insulin / P01317 (Bos Taurus)	GIVEQCCASVCSLYQLENYCN / FVNQHLCGSHLVEALYLVCGERGFFYTPKA	71
6	ubiquitin / P0CG48 (Bos Taurus)	MQIFVKTLTGKTITLEVEPSDTIENVKAKIQDKEGIPPDQQRLIFAGKQLEDGRTLSDYNIQKESTLHLVLRLRGG	75
7	cytochrome C / P00004 (Equus caballus)	GDVEKGKKIFVQKCAQCHTVEKGGKHKTGPNLHGLFGRKTGQAPGFTYTDANKNKGITWKEETLMEYLENPKKYIPGTKMI FAGIKKKTEREDLIAYLKKATNE	76
8	holo myoglobin / P68082 (Equus caballus)	MGLSDGEWQQVLNVWGKVEADIAGHGQEVLIRLFTGHPETLEKFDKFKHLKTEAEMKASEDLKKHGTVVLTALGGILKKKG HHEAELKPLAQSHATKHKIPIKYLEFISDAIIHVLHSKHPGDFGADAQGAMTKALELFRNDIAAKYKELGFQG	77
9	pepsin / P00791 (Sus scrofa)	IGDEPLENYLDTEYFGTIGIGTPAQDFTVIFDTGSSNLWVPSVYCSSLACSDHNQFNPDDSSTFEATSQELSITYGTGSMTGILGY DTVQVGGISDTNQIFGLSETEPGSFLYYAPFDGILGLAYPSISASGATPVFDNLWDQGLVSQDLFSVYLSSNDDSGSVVLLGGID SSYYTGSLNWVPVSVEGYWQITLDSITMDGETIACSGGCQAIVDTGTSLLTGPTSAIANIQSDIGASENSDGEMVISCSSIDSLPD IVFTINGVQYPLSPSAYILQDDDSCTSGFEGMDVPTSSGELWILGDVFIRQYYTVFDRANNKVGLAPVA	78
10	ovalbumin / P01012 (Gallus gallus)	GSIGAASMEFCFDVFKELKVHHANENIFYCPIAIMSALAMVYLGAKDSTRTQINKVVRFDKLPGFGDSIEAQCGTSVNVHSSLR DILNQITKPNDVYSFSLASRLYAEERYPILPEYLQCVKELYRGGLEPINFQTAADQARELINSWVESQTNGIIRNVLQPSSVDSQT AMVLVNAIVFKGLWEKAFKDEDTQAMPFRVTEQESKPVQMMYQIGLFRVASMASEKMKILELPFASGTMSMLVLLPDEVSG LEQLESIINFEKLTEWTSSNVMEERKIKVYLPRMKMEEKYNLTSVLMAMGITDVFSSSANLSGISSAESLKISQAVHAAHAEINE AGREVVGSAEAGVDAASVSEEFRADHPFLFCIKHIATNAVLFFGRCVSP	79
11	IgG-Fc / P01857 (Homo sapiens)	THTCPPCPAPELLGGPSVFLFPPKPKDTLMISRTPEVTCVVVDVSHEDPQVKFNWYVDGVQVHNAKTKPREQQYNSTYRVVS VLTVLHQNWLDGKEYKCKVSNKALPAPIEKTISKAKGQPREPQVYTLPPSREEMTKNQVSLTCLVKGFYPSDIAVEWESNGQP ENNYKTTPPVLDSDGSFFLYSKLTVDKSRWQQGNVFSCSVMHEALHNHYTQKSLSLSPG	80
12	streptavidin / P22629 (Streptomyces avidinii)	MEAGITGTWYNQLGSTFIVTAGADGALTGTYESAVGNAESRYVLTGRYDSAPATDGSGTALGWTVAWKNNYRNAHSATTW SGQYVGGAEARINTQWLLTSGTTEANAWKSTLVGHDTFTKVKPSAASIDAAKKA	82

Table S1 - continued

no.	name / code (organism) [a)]	amino acid sequence [b)]	lit. ref
13	hemoglobin / P69905 and P68871 (Homo sapiens)	VLSPADKTNVKAAWGKVGAHAGEYGAEALERMFLSFPTTKTYFPHFDLSHGSAQVKGHGKKVADALTNAVAHVDDMPNA LSALSDLHAHKLRVDPVNFKLLSHCLLVTLAAHLPAEFTPAVHASLDKFLASVSTVLTSKYR and VHLTPEEKSAVTALWGKVNVDEVGGEALGRLLVVYPWTQRFFESFGDLSTPDAVMGNPKVKAHGKKVLGAFSDGLAHLDN LKGTFATLSELHCDKLHVDPENFRLLGNVLVCVLAHHFGKEFTPPVQAAYQKVVAGVANALAHKYH	83
14	serum albumin / P02769 (Bos taurus)	DTHKSEIAHRFKDLGEEHFKGLVLIAFSQYLQQCPFDEHVKLVNELTEFAKTCVADESHAGCEKSLHTLFGDELCKVASLRET YGDMADCCEKQEPERNECFLSHKDDSPDLPKLKPDPNTLCDEFKADEKKFWGKYLYEIARRHPYFYAPELLYYANKYNGVF QECCQAEDKGACLLPKIETMREKVLTSSARQRLRCASIQKFGERALKAWSVARLSQKFPKAEFVEVTKLVTDLTKVHKECCH GDLLECADDRADLAKYICDNQDTISSKLKECCDKPLLEKSHCIAEVEKDAIPENLPPLTADFAEDKDVCKNYQEAKDAFLGSF LYEYSRRHPEYAVSVLLRLAKEYEATLEECCAKDDPHACYSTVFDKLKHLVDEPQNLIKQNCDQFEKLGEYGFQNALIVRYT RKVPQVSTPTLVEVSRSLGKVGTRCCTKPESERMPCTEDYLSLILNRLCVLHEKTPVSEKVTKCCTESLVNRRPCFSALTPDET YVPKAFDEKLFTFHADICTLPDTEKQIKKQTALVELLKHKPKATEEQLKTVMENFVAFVDKCCAADDKEACFAVEGPKLVVS TQTALA	77
15	alcohol dehydrogenase / P00330 (Saccharomyces cerevisiae)	MSIPETQKGVIFYESHGKLEYKDIPVPKPKANELLINVKYSGVCHTDLHAWHGDWPLPVKLPLVGGHEGAGVVVGMGENVK GWKIGDYAGIKWLNGSCMACEYCELGNESNCPHADLSGYTHDGSFQQYATADAVQAAHIPQGTDLAQVAPILCAGITVYKA LKSANLMAGHWVAISGAAGGLGSLAVQYAKAMGYRVLGIDGGEGKEELFRSIGGEVFIDFTKEKDIVGAVLKATDGGAHGV INVSVSEAAIEASTRYVRANGTTVLVGMPAGAKCCSDVFNQVVKSISIVGSYVGNRADTREALDFFARGLVKSPIKVVGLSTLP EIYEKMEKGQIVGRYVVDTSK	84

[a)] UniProt accession no.; species in parentheses; n.a.: not applicable [b)] single letter code; sequences may differ from those of the UniProt database

Table S2: From 3D structures computed CCS_{He} values of peptides, single-domain proteins and complexes.

no.	protein name	3D data [a)]	z	CCS_{He} [b)]
1	angiotensin II	AIIPF1	1	301
		(Pepfold4)	2	305
2	bradykinin	BrPF1	1	257
		(Pepfold4)	2	259
3	angiotensin I	AIPF1	1	392
		(Pepfold4)	2	333
4	melittin	2mlt	3	589
			4	594
			5	599
5	insulin	6qq7	3	781
			4	785
			5	791
6	ubiquitin	1ubq	4	1096
			5	1098
			6	1102
7	cytochrome c	1hrc	5	1328
			6	1330
			7	1335
			8	1340
8	holo myoglobin	1mbn	8	1750
			9	1753
9	pepsin	4pep	9	2793
			10	2795
10	ovalbumin	1ova	11	3337
			12	3340
			13	3346
11	IgG-Fc	1fc1	12	4153
			13	4153
			14	4155
12	streptavidin	1swb	15	3770
			16	3776
			17	3780
13	hemoglobin	1gzx	16	4164
			17	4769
14	serum albumin	3v03	15	4824
			16	4830
			17	4833
15	alcohol dehydrogenase	4w6z [c)]	24	8428
			25	8430
			26	8435
			27	8434

a) pdb files, unless stated otherwise b) calculated by Collidoscope c) dimers moved closer

Table S3: Experimentally determined molecular masses of peptides, single domain proteins and complexes [a].

no.	protein name	z	*m/z*	MM
1	angiotensin II	1	1046.61	1046 ± 0
		1	1046.61	
		2	523.82	
		2	523.82	
2	bradykinin	1	1060.66	1060 ± 0
		1	1060.67	
		2	530.84	
		2	530.85	
3	angiotensin I	1	1296.78	1296 ± 0
		2	648.89	
		2	648.89	
4	melittin	3	949.45	2847 ± 0
		3	949.47	
		4	712.37	
		4	712.37	
		5	570.11	
		5	570.11	
5	insulin	3	1912.25	5733 ± 1
		3	1912.25	
		4	1434.19	
		4	1434.19	
		5	1147.55	
		5	1147.55	
6	ubiquitin	4	2142.17	8565 ± 0
		4	2142.21	
		5	1713.93	
		5	1713.97	
		6	1428.45	
		6	1428.45	
		6	1428.46	
		6	1428.46	
7	cytochrome c	5	2473.12	12359 ± 1
		5	2473.14	
		6	2060.97	
		6	2061.15	
		7	1766.45	
		7	1766.58	
		8	1545.66	
		8	1545.66	
8	holo myoglobin	8	2196.44	17564 ± 1
		8	2196.42	
		9	1952.60	
		9	1952.47	
9	pepsin	9	3844.33	34589 ± 1
		9	3844.11	
		10	3460.01	
		10	3459.96	

Table S3 - continued

no.	protein name	z	*m/z*	MM
10	ovalbumin	11	4016.92	44176 ± 1
		11	4017.11	
		12	3682.22	
		12	3682.54	
		13	3399.14	
		13	3399.22	
11	IgG-Fc	12	4430.58	53155 ± 2
		12	4430.58	
		13	4089.70	
		13	4090.01	
		14	3797.59	
		14	3797.86	
12	streptavidin	15	3742.09	56117 ± 1
		15	3742.19	
		16	3508.31	
		16	3508.34	
		17	3301.96	
		17	3302.11	
13	hemoglobin	16	4043.23	64715 ± 33
		16	4047.50	
		16	4047.25	
		16	4049.41	
		16	4045.70	
		17	3804.91	
		17	3808.14	
		17	3806.17	
		17	3807.65	
		17	3807.41	
14	serum albumin	15	4432.04	66469 ± 4
		15	4432.59	
		16	4155.21	
		16	4155.07	
		17	3910.85	
		17	3911.10	
15	alcohol dehydrogenase	24	6151.25	147668 ± 43
		24	6154.87	
		24	6157.13	
		25	5907.41	
		25	5909.42	
		26	5909.32	
		26	5679.34	
		26	5680.55	
		26	5681.01	
		27	5468.29	
		27	5470.39	
		27	5468.42	

a) duplicate measurements

Table S4: Experimentally determined drift times, $^{TW}CCS_{N2}$ values, and literature reference values of peptides, single domain proteins, and complexes.

no.	protein name	experimental				$^{TW}CCS_{N2}$ statistics			literature a)	
		z	*m/z*	dt	$^{TW}CCS_{N2}$	average	std.dev.	n	CCS_{N2}	ref.
1	angiotensin II	1	1046.61	11.75	241	242	2	2	n.d.	n.d.
		1	1046.61	12.03	244	/	/	/	n.d.	n.d.
		2	523.82	4.70	334	334	0	2	**335**	96
		2	523.82	4.70	334	/	/	/	355	57
2	bradykinin	1	1060.66	11.89	242	243	1	2	245	101
		1	1060.67	12.03	244	/	/	/	n.d.	n.d.
		2	530.84	4.56	331	331	0	2	344	96
		2	530.85	4.56	331	/	/	/	348	57
3	angiotensin I	1	1296.78	14.65	271	/	/	/	n.d.	n.d.
		2	648.89	5.53	351	351	0	2	n.d.	n.d.
		2	648.89	5.53	351	/	/	/	n.d.	n.d.
4	melittin	3	949.45	8.16	605	605	0	2	n.d.	n.d.
		3	949.47	8.16	605	/	/	/	n.d.	n.d.
		4	712.37	5.53	698	698	0	2	n.d.	n.d.
		4	712.37	5.53	698	/	/	/	n.d.	n.d.
		5	570.11	4.70	829	833	5	2	n.d.	n.d.
		5	570.11	4.84	836	/	/	/	n.d.	n.d.
5	insulin	3	1912.25	12.30	732	734	3	2	729	95
		3	1912.25	12.44	736	/	/	/	732	95
		4	1434.19	8.43	815	818	4	2	865	95
		4	1434.19	8.57	821	/	/	/	868	95
		5	1147.55	7.19	955	955	0	2	1038	95
		5	1147.55	7.19	955	/	/	/	1038	95
6	ubiquitin	4	2142.17	11.75	952	952	0	2	956	95
		4	2142.21	11.75	952	/	/	/	956	95
		4	/	/	/	/	/	/	1180	100
		4	/	/	/	/	/	/	**949**	97
		4	/	/	/	/	/	/	1116	97
		4	/	/	/	/	/	/	1118	98
		5	1713.93	9.95	1097	1100	5	2	1132	95
		5	1713.97	10.09	1104	/	/	/	1137	95
		5	/	/	/	/	/	/	1310	100
		5	/	/	/	/	/	/	**1011**	97
		5	/	/	/	/	/	/	1221	97
		5	/	/	/	/	/	/	1167	98
		5	/	/	/	/	/	/	1208	98
		5	/	/	/	/	/	/	1154	30
		6	1428.45	8.85	1248	1253	6	2	1046	101
		6	1428.45	11.20	1394	1403	12	2	1220	101
		6	1428.46	8.99	1257	/	/	/	1200	57
		6	1428.46	11.47	1411	/	/	/	1313	95
		6	/	/	/	/	/	/	1411	95
		6	/	/	/	/	/	/	1730	100
		6	/	/	/	/	/	/	1229	98
		6	/	/	/	/	/	/	1268	98
		6	/	/	/	/	/	/	1382	98
		6	/	/	/	/	/	/	1214	30

Table S4 - continued

no.	protein name	experimental				$^{TW}CCS_{N2}$ statistics			literature	
		z	*m/z*	dt	$^{TW}CCS_{N2}$	average	std.dev.	n	CCS_{N2}	ref.
7	cytochrome c	5	2473.12	12.03	1203	1200	5	2	1204	95
		5	2473.14	11.89	1196	/	/	/	1199	95
		5	/	/	/	/	/	/	1146	97
		5	/	/	/	/	/	/	1393	97
		6	2060.97	10.37	1341	1346	6	2	**1490**	96
		6	2061.15	10.51	1350	/	/	/	1376	95
		6	/	/	/	/	/	/	1381	95
		6	/	/	/	/	/	/	1477	97
		6	/	/	/	/	/	/	1360	97
		6	/	/	/	/	/	/	1454	98
		7	1766.45	11.20	1625	1630	7	2	1247	101
		7	1766.58	11.34	1635	/	/	/	**1590**	96
		7	/	/	/	/	/	/	1645	95
		7	/	/	/	/	/	/	1652	95
		7	/	/	/	/	/	/	1537	98
		8	1545.66	10.23	1778	1783	8	2	1845	101
		8	1545.66	10.37	1789	/	/	/	2061	101
		8	/	/	/	/	/	/	1827	95
		8	/	/	/	/	/	/	1835	95
		8	/	/	/	/	/	/	1654	98
		8	/	/	/	/	/	/	1887	98
		8	/	/	/	/	/	/	2043	98
		8	/	/	/	/	/	/	2201	98
8	holo myoglobin	8	2196.44	11.06	1962	1964	3	2	1673	101
		8	2196.42	11.20	1966	/	/	/	1872	95
		8	/	/	/	/	/	/	1879	95
		8	/	/	/	/	/	/	**1937**	97
		8	/	/	/	/	/	/	1949	98
		9	1952.60	10.09	2174	2176	3	2	1785	101
		9	1952.47	10.23	2179	/	/	/	2045	95
		9	/	/	/	/	/	/	2054	95
		9	/	/	/	/	/	/	**2085**	97
		9	/	/	/	/	/	/	2003	98
		9	/	/	/	/	/	/	2114	98
9	pepsin	9	3844.33	17.00	2407	2410	4	2	n.d.	n.d.
		9	3844.11	17.14	2412	/	/	/	n.d.	n.d.
		10	3460.01	14.65	2586	2589	4	2	n.d.	n.d.
		10	3459.96	14.79	2591	/	/	/	n.d.	n.d.
10	ovalbumin	11	4016.92	18.25	2993	2985	12	2	n.d.	n.d.
		11	4017.11	17.83	2976	/	/	/	n.d.	n.d.
		12	3682.22	15.76	3153	3150	4	2	n.d.	n.d.
		12	3682.54	15.62	3147	/	/	/	n.d.	n.d.
		13	3399.14	14.10	3334	3334	0	2	n.d.	n.d.
		13	3399.22	14.10	3334	/	/	/	n.d.	n.d.
11	IgG-Fc	12	4430.58	19.77	3334	3325	13	2	n.d.	n.d.
		12	4430.58	19.35	3315	/	/	/	n.d.	n.d.
		13	4089.70	17.42	3497	3487	15	2	n.d.	n.d.
		13	4090.01	17.00	3476	/	/	/	n.d.	n.d.
		14	3797.59	14.93	3634	3634	0	2	n.d.	n.d.
		14	3797.86	14.93	3634	/	/	/	n.d.	n.d.

Table S4 - continued

no.	protein name	experimental				$^{TW}CCS_{N2}$ statistics			literature	
		z	*m/z*	dt	$^{TW}CCS_{N2}$	average	std.dev.	n	CCS_{N2}	ref.
12	streptavidin	15	3742.09	16.31	3972	3964	11	2	3890	100
		15	3742.19	16.04	3957	/	/	/	3884	99
		16	3508.31	14.65	4137	4133	6	2	3949	99
		16	3508.34	14.52	4129	/	/	/	n.d.	n.d.
		17	3301.96	12.58	4263	4267	6	2	n.d.	n.d.
		17	3302.11	12.72	4272	/	/	/	n.d.	n.d.
13	hemoglobin	16	4043.23	18.04	4341	/	/	/	4300	99
		16	4047.50	17.50	4308	4311	5	4	n.d.	n.d.
		16	4047.25	17.50	4308	/	/	/	n.d.	n.d.
		16	4049.41	17.68	4319	/	/	/	n.d.	n.d.
		16	4045.70	17.50	4308	/	/	/	n.d.	n.d.
		17	3804.91	17.50	4578	/	/	/	4500	99
		17	3808.14	16.95	4542	4540	6	4	n.d.	n.d.
		17	3806.17	16.95	4542	/	/	/	n.d.	n.d.
		17	3807.65	16.95	4542	/	/	/	n.d.	n.d.
		17	3807.41	16.77	4531	/	/	/	n.d.	n.d.
14	serum albumin	15	4432.04	18.80	4112	4112	0	2	**4490**	96
		15	4432.59	18.80	4112	/	/	/	4310	95
		15	/	/	/	/	/	/	4310	95
		15	/	/	/	/	/	/	4450	100
		15	/	/	/	/	/	/	4548	98
		16	4155.21	17.56	4312	4308	6	2	**4470**	96
		16	4155.07	17.42	4303	/	/	/	4460	95
		16	/	/	/	/	/	/	4460	95
		16	/	/	/	/	/	/	4510	100
		16	/	/	/	/	/	/	4567	98
		17	3910.85	17.28	4563	4572	13	2	**4490**	96
		17	3911.10	17.56	4581	/	/	/	4706	95
		17	/	/	/	/	/	/	4740	95
		17	/	/	/	/	/	/	4590	100
		17	/	/	/	/	/	/	4648	98
15	alcohol dehyrogenase	24	6151.25	27.88	7400	7411	19	3	**7450**	96
		24	6154.87	27.88	7400	/	/	/	7540	100
		24	6157.13	28.25	7433	/	/	/	7693	98
		25	5907.41	26.43	7571	7577	26	3	**7440**	96
		25	5909.42	26.24	7553	/	/	/	7570	100
		25	5909.32	26.79	7605	/	/	/	7750	98
		26	5679.34	24.6	7695	7695	18	3	**7500**	96
		26	5680.55	24.42	7677	/	/	/	7814	98
		26	5681.01	24.79	7713	/	/	/	n.d.	n.d.
		27	5468.29	23.15	7843	7824	19	3	n.d.	n.d.
		27	5470.39	22.96	7824	/	/	/	n.d.	n.d.
		27	5468.42	22.78	7806	/	/	/	n.d.	n.d.

a) n.d.: not determined; n.a.: not applicable

b) bold printed values were used for calibration

Table S5: Calculated and experimentally determined CCSs of peptides, proteins, and complexes.

no.	protein name	z	$^{TW}CCS_{N2}$ [Å^2] a)	CCS_{He} [Å^2]
1	angiotensin II	2	334 ± 0	305
2	Bradykinin	2	331 ± 0	259
3	angiotensin I	2	351 ± 0	333
4	Melittin	4	698 ± 0	594
5	Insulin	4	818 ± 4	785
6	Ubiquitin	5	1100 ± 5	1098
7	cytochrome c	7	1630 ± 7	1335
8	holo myoglobin	8	1964 ± 3	1750
9	Pepsin	10	2589 ± 4	2795
10	Ovalbumin	12	3150 ± 4	3337
11	IgG-Fc	13	3487 ± 15	4153
12	Streptavidin	16	4133 ± 6	3776
13	Hemoglobin	17	4540 ± 6	4769
14	serum albumin	17	4572 ± 13	4833
15	alcohol dehydrogenase	25	7577 ± 26	8430

a) duplicate measurements

Table S6: Amino acid sequences of multi-domain proteins.

no.	name / code (organism) [a)]	amino acid sequence [b)]	lit. ref
A1	protein G'e / Q54181 (Streptococcus sp.)	GSSHHHHHHSSGLVPRGSHMASMTGGQQMGRDPNSSSVDKLAAALETYKLILNGKTLKGETTTEAVDAATAEKVFKQYAND<br>NGVDGEWTYDDATKTFTVTEKPEVIDASELTPAVTTYKLVINGKTLKGETTTEAVDAATAEKVFKQYANDNGVDGEWTYDDA<br>TKTFTVTEKPEVIDASELTPAVTTYKLVINGKTLKGETTTKAVDAETAEKAFKQYANDNGVDGVWTYDDATKTFTVTE	85
A2	IgG1 / P01837 and P01869 (Mus musculus)	RADAAPTVSIFPPSSEQLTSGGASVVCFLNNFYPKDINVKWKIDGSERQNGVLNSWTDQDSKDSTYSMSSTLTLTKDEYERHNS<br>YTCEATHKTSTSPIVKSFNRNEC<br>and<br>AKTTPPSVYPLAPGSAAQTNSMVTLGCLVKGYFPEPVTVTWNSGSLSSGVHTFPAVLQSDLYTLSSSVTVPSSPRPSETVTCNVA<br>HPASSTKVDKKIVPRDCGCKPCICTVPEVSSVFIFPPKPKDVLTITLTPKVTCVVVDISKDDPEVQFSWFVDDVEVHTAQTQPREE<br>QFNSTFRSVSELPIMHQDWLNGKEFKCRVNSAAFPAPIEKTISKTKGRPKAPQVYTIPPPKEQMAKDKVSLTCMITDFFPEDITVE<br>WQWNGQPAENYKNTQPIMNTNGSYFVYSKLNVQKSNWEAGNTFTCSVLHEGLHNHHTEKSLSHSPGLQLDETCAEAQDGEL<br>DGLWTTITIFISLFLLSVCYSAAVTLFKVKWIFSSVVELKQTLVPEYKNMIGQAP	34

[a)] UniProt accession no.; species in parentheses; n.a.: not applicable

[b)] single letter code; sequences may differ from those of the UniProt database

Table S7: Experimentally determined molecular masses of multi-domain proteins [a)].

no.	protein name	z	*m/z*	MM
A1	protein G'e	8	3251.03	26001 ± 1
		8	3251.08	
		8	3251.21	
		8	3251.26	
		9	2889.94	
		9	2889.99	
		9	2890.04	
		9	2890.08	
		10	2601.06	
		10	2601.13	
		10	2601.10	
		10	2601.10	
		11	2364.73	
		11	2364.62	
		11	2364.77	
		11	2364.77	
		12	2167.66	26001 ± 1
		12	2167.68	
		13	2001.06	
		13	2001.10	
		14	1858.13	
		14	1858.23	
		15	1734.25	
		15	1734.40	
		16	1625.99	
		16	1626.06	
		17	1530.52	
		17	1530.44	
		18	1445.56	
		18	1445.51	
		19	1369.46	
		19	1369.52	
A2γ	antiMSP1_{19}	24	6270.78	150514 ± 50
		25	6024.48	
		26	5789.58	
		27	5574.77	
		36	4164.22	149758 ± 97
		37	4052.94	
		38	3942.48	
		39	3838.78	
		40	3742.06	
		41	3655.22	
		42	3565.99	
		43	3482.05	
		44	3406.13	
		45	3327.73	
		46	3257.03	
		47	3186.87	
		48	3124.22	
		49	3054.31	
		50	2996.77	

a) duplicate measurements

Table S8: Experimentally determined drift times, $^{TW}CCS_{N2}$ values, and literature reference values of compacted and extended "native" protein G'e.

no.	protein name (conformation)	experimental				$^{TW}CCS_{N2}$ statistics			lit. ref. a)	
		z	*m/z*	dt	$^{TW}CCS_{N2}$	average	std.dev.	n	CCS_{N2}	ref.
A1I	protein G'e	8	3251.03	14.79	2073	2079	4	4	2540	91
		8	3251.08	14.93	2078	/	/	/	n.d.	n.d.
		8	3251.21	15.07	2082	/	/	/	n.d.	n.d.
		8	3251.26	15.07	2082	/	/	/	n.d.	n.d.
		9	2889.94	12.58	2258	2260	2	4	2422	91
		9	2889.99	12.58	2258	/	/	/	n.d.	n.d.
		9	2890.04	12.72	2262	/	/	/	n.d.	n.d.
		9	2890.08	12.72	2262	/	/	/	n.d.	n.d.
		10	2601.06	10.92	2446	2446	4		2445	91
		10	2601.13	11.06	2451	/	/	/	n.d.	n.d.
		10	2601.10	10.78	2441	/	/	/	n.d.	n.d.
		10	2601.10	10.92	2446	/	/	/	n.d.	n.d.
A1II	protein G'e	13	2001.06	10.37	3153	3153	0	2	n.d.	n.d.
		13	2001.10	10.37	3153	/	/	/	n.d.	n.d.
		14	1858.13	10.51	3403	3403	0	2	n.d.	n.d.
		14	1858.23	10.51	3403	/	/	/	n.d.	n.d.
		15	1734.25	10.37	3638	3686	0	2	n.d.	n.d.
		15	1734.40	10.37	3638	/	/	/	n.d.	n.d.
		16	1625.99	10.92	3914	3919	6	2	n.d.	n.d.
		16	1625.99	12.03	3981	3986	6	2	n.d.	n.d.
		16	1626.06	11.06	3923	/	/	/	n.d.	n.d.
		16	1626.06	12.17	3990	/	/	/	n.d.	n.d.
		17	1530.52	10.51	4133	4113	0	2	n.d.	n.d.
		17	1530.52	11.2	4177	4182	6	2	n.d.	n.d.
		17	1530.44	10.51	4133	/	/	/	n.d.	n.d.
		17	1530.44	11.34	4186	/	/	/	n.d.	n.d.
		18	1445.56	10.65	4386	4390	6	2	n.d.	n.d.
		18	1445.51	10.78	4394	/	/	/	n.d.	n.d.
		19	1369.46	10.23	4599	4604	7	2	n.d.	n.d.
		19	1369.46	11.34	4679	/	/	/	n.d.	n.d.
		19	1369.52	10.37	4609	/	/	/	n.d.	n.d.

a) n.d.: not determined

Table S9: Experimentally determined drift times and $^{TW}CCS_{N2}$ values for denatured protein G'e.

no.	protein name (conformation)	z	experimental			$^{TW}CCS_{N2}$ statistics		
			m/z	dt	$^{TW}CCS_{N2}$	average	std.dev.	n
A1III	protein G'e (denatured)	17	1530.27	11.48	4195	4195	0	2
		17	1530.27	13.49	4324	4324	0	2
		17	1530.37	11.48	4195	/	/	/
		17	1530.37	13.49	4324	/	/	/
		18	1445.34	10.75	4392	4399	9	2
		18	1445.34	12.58	4517	4517	0	2
		18	1445.37	10.94	4405	/	/	/
		18	1445.37	12.58	4517	/	/	/
		19	1369.32	10.21	4598	4605	9	2
		19	1369.32	11.85	4715	4715	0	2
		19	1369.37	10.39	4611	/	/	/
		19	1369.37	11.85	4715	/	/	/
		20	1300.90	11.30	4922	4922	0	2
		20	1300.92	11.30	4922	/	/	/
		21	1239.06	10.75	5125	5125	0	2
		21	1239.09	10.75	5125	/	/	/
		22	1182.74	10.39	5339	5339	0	2
		22	1182.80	10.39	5339	/	/	/
		23	1131.43	9.84	5534	5534	0	2
		23	1131.30	9.84	5534	/	/	/
		24	1084.24	9.48	5742	5742	0	2
		24	1084.33	9.48	5742	/	/	/
		25	1040.88	9.11	5947	5947	0	2
		25	1040.94	9.11	5947	/	/	/
		26	1000.97	8.93	6167	6167	0	2
		26	1000.96	8.93	6167	/	/	/
		27	963.93	8.57	6367	6367	0	2
		27	963.90	8.57	6367	/	/	/
		28	929.51	8.38	6583	6583	0	2
		28	929.49	8.38	6583	/	/	/
		29	897.49	8.20	6799	6799	0	2
		29	897.50	8.20	6799	/	/	/
		30	867.63	7.84	6993	7003	14	2
		30	867.61	8.02	7013	/	/	/
		31	839.62	7.84	7226	7226	0	2
		31	839.66	7.84	7226	/	/	/
		32	813.44	7.65	7436	7436	0	2
		32	813.40	7.65	7436	/	/	/

Table S10: From 3D structures computed CCS_{He} values of multi-domain proteins.

no. [a)]	protein name	3D data [b)]	conformation	z	CCS_{He} [c)]
A1I	protein G'e	I-TASSER (o-shaped)	compact	9	2673
A1I'	protein G'e	I-TASSER (o-shaped)	compact	14	2673
A1II	protein G'e	AlphaFold 3 (I-shaped)	extended	9	4020
A1II'	protein G'e	AlphaFold 3 (I-shaped)	extended	14	3969
A2xI	IgG1	Hojrup model (m-shaped)	compact	25	7291
A2xI’	IgG1	Hojrup model (m-shaped)	compact	26	7295
A2xI'’	IgG1	Hojrup model (m-shaped)	compact	37	7332
A2xII	IgG1	1igy (Y-shaped)	extended	25	10009
A2xII’	IgG1	1igy (Y-shaped)	extended	26	10005
A2xII’’	IgG1	1igy (Y-shaped)	extended	37	9948
A2xII’"	IgG1	1igy (Y-shaped)	extended	38	9939
A2xII’’’’	IgG1	1igy (Y-shaped)	extended	39	9935

a) I: compacted; II: extended

b) pdb files, unless stated otherwise

c) calculated by Collidoscope

Table S11: Experimentally determined drift times, $^{TW}CCS_{N2}$ values, and literature reference values of compacted and extended "native" antibodies.

no.	protein name	experimental				$^{TW}CCS_{N2}$ statistics			lit. ref. a)	
	(conformation)	z	*m/z*	dt	$^{TW}CCS_{N2}$	average	std.dev.	n	CCS_{N2}	ref. a)
A2αI	HAM1101 (compact)	24	6296.56	28.43	7449	7491	59	2	6590	62
		24	6274.03	29.35	7533	/	/	/	7700	61
		24	/	/	/	/	/	/	7276	59
		25	6052.26	26.79	7605	7605	0	2	7170	62
		25	6020.74	26.79	7605	/	/	/	8000	61
		25	/	/	/	/	/	/	7246	59
		26	5819.61	25.51	7784	7722	88	2	7610	62
		26	5788.81	24.24	7659				7247	61
		27	5596.44	24.24	7954	7917	52	2	7292	59
		27	5573.74	23.51	7880	/	/	/	7292	59
A2αII	HAM1101 (extended)	37	4065.49	18.59	10114	10127	18	2	n.d.	n.d.
		37	4061.52	18.77	10139	/	/	/	n.d.	n.d.
		38	3959.08	18.04	10308	10308	0	1	n.d.	n.d.
		38	/	/	/	/	/	/	n.d.	n.d.
		39	3859.55	17.13	10446	10473	38	2	n.d.	n.d.
		39	3854.31	17.50	10500	/	/	/	n.d.	n.d.
		40	/	/	/	/	/	/	n.d.	n.d.
		40	/	/	/	/	/	/	n.d.	n.d.
A2βI	αMSP1$_{19}$ (compact)	24	6276.37	28.25	7433	7408	35	2	6590	62
		24	6270.64	27.70	7383	/	/	/	7700	61
		24	/	/	/	/	/	/	7276	59
		25	6025.09	25.70	7502	7494	12	2	7170	62
		25	6016.05	25.52	7485	/	/	/	8000	61
		25	/	/	/	/	/	/	7246	59
		26	5779.56	24.44	7679	7687	11	2	7610	62
		26	5788.98	24.60	7695	/	/	/	7247	61
		27	5565.11	24.06	7936	7945	13	2	7292	59
		27	5575.75	24.24	7954	/	/	/	7292	59
A2βII	αMSP1$_{19}$ (extended)	37	4052.72	18.77	10139	10152	18	2	n.d.	n.d.
		37	4053.58	18.95	10164	/	/	/	n.d.	n.d.
		38	3940.34	18.77	10413	10334	112	2	n.d.	n.d.
		38	3943.13	17.67	10255	/	/	/	n.d.	n.d.
		39	3840.16	18.40	10633	10539	133	2	n.d.	n.d.
		39	3838.62	17.12	10445	/	/	/	n.d.	n.d.
		40	3742.97	17.50	10770	10824	76	2	n.d.	n.d.
		40	3741.77	18.21	10877	/	/	/	n.d.	n.d.
A2γI	αHis-tag (compact)	24	6213.26	26.97	7317	7317	0	2	6590	62
		24	6213.40	26.97	7317	/	/	/	7700	61
		24	/	/	/	/	/	/	7276	59
		25	5962.83	25.87	7518	7519	1	2	7170	62
		25	5963.27	25.88	7519	/	/	/	8000	61
		25	/	/	/	/	/	/	7246	59
		26	5735.50	24.60	7695	7696	1	2	7610	62
		26	5736.72	24.61	7696	/	/	/	7247	61
		27	5525.57	24.04	7934	7944	14	2	7292	59
		27	5519.16	24.24	7954	/	/	/	7292	59

Table S11: continued

no.	protein name (conformation)	experimental				$^{TW}CCS_{N2}$ statistics			lit. ref. a)	
		z	*m/z*	dt	$^{TW}CCS_{N2}$	average	std.dev.	n	CCS_{N2}	ref. a)
A2γII	αHis-tag (extended)	37	4028.55	18.23	10064	10089	35	2	n.d.	n.d.
		37	4030.35	18.59	10114	/	/	/	n.d.	n.d.
		38	3920.53	17.32	10205	10235	42	2	n.d.	n.d.
		38	3926.81	17.73	10264	/	/	/	n.d.	n.d.
		39	3823.52	17.13	10446	10420	37	2	n.d.	n.d.
		39	3822.44	16.77	10393	/	/	/	n.d.	n.d.
		40	3726.04	16.22	10577	10591	19	2	n.d.	n.d.
		40	3725.00	16.40	10604	/	/	/	n.d.	n.d.
A2δI	Rituximab (compact)	24	6164.63	27.34	7351	7326	35	2	6590	62
		24	6170.24	26.79	7301	/	/	/	7700	61
		24	/	/	/	/	/	/	7276	59
		25	5921.83	25.88	7519	7519	0	2	7170	62
		25	5916.86	25.88	7519	/	/	/	8000	61
		25	/	/	/	/	/	/	7246	59
		26	5690.55	24.60	7695	7695	0	2	7610	62
		26	5688.94	24.60	7695	/	/	/	7247	61
		27	5476.48	23.51	7880	7880	0	2	7292	59
		27	5477.45	23.35	7880	/	/	/	7292	59
		28	5280.80	22.80	8097	8097	0	2	n.d.	n.d.
		28	5281.53	22.60	8097	/	/	/	n.d.	n.d.
A2δII	Rituximab (extended)	37	3989.21	18.04	10037	10087	70	2	n.d.	n.d.
		37	3992.19	18.75	10136	/	/	/	n.d.	n.d.
		38	3884.50	18.77	10413	10322	129	2	n.d.	n.d.
		38	3886.32	17.50	10231	/	/	/	n.d.	n.d.
		39	3785.57	17.30	10471	10460	16	2	n.d.	n.d.
		39	3786.70	17.15	10449	/	/	/	n.d.	n.d.
		40	3690.97	16.95	10687	10714	38	2	n.d.	n.d.
		40	3692.03	17.31	10741	/	/	/	n.d.	n.d.

a) n.d.: not determined

Table S12: Experimentally determined drift times and $^{TW}CCS_{N2}$ values for denatured IgG1.

no.	protein name	experimental				$^{TW}CCS_{N2}$ statistics		
		z	*m/z*	dt	$^{TW}CCS_{N2}$	average	std.dev.	n
A2αIII	HAM1101	43	3419.21	14.94	11163	11253	127	2
		43	3416.71	16.05	11343	/	/	/
		44	3342.72	16.40	11665	11520	205	2
		44	3340.96	14.65	11375	/	/	/
		45	3270.07	15.31	11745	11903	223	2
		45	3267.83	17.17	12061	/	/	/
		46	3199.45	16.22	12164	12054	156	2
		46	3197.87	14.95	11944	/	/	/
		47	3132.63	16.02	12394	12397	4	2
		47	3130.70	16.05	12399	/	/	/
		48	3068.05	14.22	12331	12529	279	2
		48	3066.94	16.40	12726	/	/	/
		49	3007.00	12.39	12250	12404	217	2
		49	3005.09	14.05	12557	/	/	/
		50	2948.57	13.67	12742	12673	98	2
		50	2946.37	12.94	12604	/	/	/
		51	2891.34	13.49	12962	13130	237	2
		51	2890.12	15.23	13297	/	/	/
		52	2835.75	18.45	14190	13793	562	2
		52	2834.45	14.40	13395	/	/	/
		53	2783.84	13.30	13433	13378	78	2
		53	2781.86	12.75	13323	/	/	/
A2βIII	αMSP1$_{19}$ (denatured)	45	3252.62	15.32	11747	11731	23	2
		45	3254.51	15.13	11715			
		46	3184.75	15.85	12100	12085	22	2
		46	3187.33	15.67	12069			
		47	3118.81	15.49	12300	12285	22	2
		47	3120.10	15.32	12269			
		48	3052.86	13.85	12264	12380	164	2
		48	3054.81	15.13	12496			
		49	2991.62	14.77	12690	12769	111	2
		49	2993.34	15.62	12847			
		50	2933.17	13.31	12674	12692	25	2
		50	2936.58	13.50	12710			
		51	2874.84	15.32	13314	13313	1	2
		51	2875.54	15.31	13312			
		52	2820.19	14.95	13503	13482	30	2
		52	2826.37	14.73	13460			
		53	2768.95	14.58	13689	13653	51	2
		53	2771.11	14.22	13617			
		54	2719.21	14.75	13984	13984	0	2
		54	2719.48	14.76	13984			

Table S12: continued

no.	protein name	experimental				$^{TW}CCS_{N2}$ statistics		
		z	*m/z*	dt	$^{TW}CCS_{N2}$	average	std.dev.	n
A2γIII	αHis-tag	43	3465.64	14.94	11163	11238	105	2
		43	3465.87	15.86	11312	/	/	/
		44	3385.56	14.75	11391	11437	64	2
		44	3387.92	15.30	11482	/	/	/
		45	3309.58	14.22	11560	11668	153	2
		45	3312.02	15.49	11776	/	/	/
		46	3238.29	16.04	12133	12071	88	2
		46	3238.98	15.32	12008	/	/	/
		47	3169.98	14.22	12074	12219	204	2
		47	3167.42	15.85	12363	/	/	/
		48	3104.99	15.56	12574	12584	14	2
		48	3103.80	15.67	12594	/	/	/
		49	3040.78	15.31	12790	12790	0	2
		49	3041.45	15.31	12790	/	/	/
		50	2979.64	15.67	13119	12931	267	2
		50	2978.04	13.67	12742	/	/	/
		51	2921.90	13.64	12991	12944	66	2
		51	2920.34	13.15	12897	/	/	/
		52	2863.60	13.15	13150	13201	72	2
		52	2862.58	13.67	13252	/	/	/
		53	2807.45	14.75	13723	13743	28	2
		53	2809.76	14.95	13763	/	/	/
A2δIII	Rituximab	43	3430.28	16.95	11489	11445	63	2
		43	3427.65	16.40	11400	/	/	/
		44	3351.05	16.58	11695	11647	68	2
		44	3350.72	16.00	11599	/	/	/
		45	3274.69	16.40	11930	11791	197	2
		45	3274.66	14.76	11652	/	/	/
		46	3203.30	16.04	12133	12073	85	2
		46	3203.55	15.35	12013	/	/	/
		47	3134.12	15.67	12331	12477	206	2
		47	3134.07	17.31	12622	/	/	/
		48	3068.08	15.31	12529	12760	327	2
		48	3068.34	17.86	12991	/	/	/
		49	3006.97	15.13	12757	12907	211	2
		49	3006.40	16.75	13056	/	/	/
		50	2946.60	14.76	12947	13017	99	2
		50	2946.96	15.50	13087	/	/	/
		51	2888.95	14.76	13207	13365	223	2
		51	2888.67	16.40	13522	/	/	/
		52	2833.61	14.58	13430	13713	400	2
		52	2833.40	14.40	13995	/	/	/
		53	2779.38	14.94	13761	13689	102	2
		53	2778.99	14.22	13617	/	/	/

Table S13: Experimentally determined drift times and $^{TW}CCS_{N2}$ values for dimeric αHis-tag.

no.	protein name	experimental				$^{TW}CCS_{N2}$ statistics		
		z	*m/z*	dt	$^{TW}CCS_{N2}$	average	std.dev.	n
A2γIV	αHis-tag	70	4256.70	19.32	19325	19277	69	2
		70	4259.12	18.95	19228			
		71	4193.41	19.14	19553	19553	0	2
		71	4202.57	19.14	19553			
		72	4139.41	19.13	19826	19730	136	2
		72	4137.25	18.42	19633			
		73	4085.59	18.41	19086	19953	0	1
		73	4082.14	18.59	19953			
		74	4028.55	18.23	20126	20177	71	2
		74	4030.35	18.59	20227			
		75	3978.76	17.50	20192	20218	36	2
		75	3973.23	17.68	20243			
		76	3920.53	17.32	20409	20468	83	2
		76	3926.81	17.73	20527			
		77	3870.92	17.12	20620	20676	78	2
		77	3872.63	17.50	20731			
		78	3823.52	17.13	20891	20838	75	2
		78	3822.44	16.77	20785			
		79	3771.90	16.95	21105	21023	116	2
		79	3774.15	16.40	20941			
		80	3726.04	16.22	21152	21180	39	2
		80	3725.00	16.40	21207			
